# Multiscale Asymptotic Analysis Reveals How Cell Growth and Subcellular Compartments Affect Tissue-Scale Hormone Transport

**DOI:** 10.1007/s11538-023-01199-4

**Published:** 2023-09-13

**Authors:** K. B. Kiradjiev, L. R. Band

**Affiliations:** 1 School of Mathematical Sciences, University of Nottingham, University Park, Nottingham, NG7 2RD UK; 2 Division of Plant and Crop Sciences, School of Biosciences, University of Nottingham, Sutton Bonington Campus, Loughborough, LE12 5RD UK

**Keywords:** Hormone transport, Multiscale analysis, Subcellular compartment, Reaction–advection–diffusion equation, Cell growth and division

## Abstract

Determining how cell-scale processes lead to tissue-scale patterns is key to understanding how hormones and morphogens are distributed within biological tissues and control developmental processes. In this article, we use multiscale asymptotic analysis to derive a continuum approximation for hormone transport in a long file of cells to determine how subcellular compartments and cell growth and division affect tissue-scale hormone transport. Focusing our study on plant tissues, we begin by presenting a discrete multicellular ODE model tracking the hormone concentration in each cell’s cytoplasm, subcellular vacuole, and surrounding apoplast, represented by separate compartments in the cell-file geometry. We allow the cells to grow at a rate that can depend both on space and time, accounting for both cytoplasmic and vacuolar expansion. Multiscale asymptotic analysis enables us to systematically derive the corresponding continuum model, obtaining an effective reaction–advection–diffusion equation and revealing how the effective diffusivity, effective advective velocity, and the effective sink term depend on the parameters in the cell-scale model. The continuum approximation reveals how subcellular compartments, such as vacuoles, can act as storage vessels, that significantly alter the effective properties of hormone transport, such as the effective diffusivity and the induced effective velocity. Furthermore, we show how cell growth and spatial variance across cell lengths affect the effective diffusivity and the induced effective velocity, and how these affect the tissue-scale hormone distribution. In particular, we find that cell growth naturally induces an effective velocity in the direction of growth, whereas spatial variance across cell lengths induces effective velocity due to the presence of an extra compartment, such as the apoplast and the vacuole, and variations in the relative sizes between the compartments across the file of cells. It is revealed that hormone transport is faster across cells of decreasing lengths than cells with increasing lengths. We also investigate the effect of cell division on transport dynamics, assuming that each cell divides as soon as it doubles in size, and find that increasing the time between successive cell divisions decreases the growth rate, which enhances the effect of cell division in slowing hormone transport. Motivated by recent experimental discoveries, we discuss particular applications for transport of gibberellic acid (GA), an important growth hormone, within the *Arabidopsis* root. The model reveals precisely how membrane proteins that mediate facilitated GA transport affect the effective tissue-scale transport. However, the results are general enough to be relevant to other plant hormones, or other substances that are transported in a similar way in any type of cells.

## Introduction

Within biological tissues, tissue-scale patterns of hormones and morphogens emerge from processes at the cell scale. Understanding how these tissue-scale patterns arise is often key to revealing how regulation of transport or metabolism at the cell scale affects developmental patterning and thus, phenotype. To understand how effective tissue-scale properties depend on processes at the cell scale, multiscale asymptotic methods enable us to derive continuum approximations of the tissue-scale dynamics; this analysis reveals precisely how the tissue-scale dynamics relate to the cell-scale processes and parameters. In this study, we consider transport within a single cell file and use multiscale asymptotics to analyse how the presence of subcellular compartments, cell growth, and cell division affect the effective tissue-scale transport. While the results could be easily adapted to study the transport of other substances within other biological tissues, we focus specifically on hormone transport within plant root tissues, which have a regular multicellular structure and uni-directional growth and so are ideally suited to these asymptotic techniques.

### Biological Background on Plant Hormones

Plant hormones play a key role in regulating growth, development, and cell division (Davies 1987). Examples include gibberellic acid (GA), abscisic acid (ABA), salicylic acid (SA), auxin and others. Dynamic hormone distributions govern plant development, for instance, controlling the sizes of developmental zones, specifying cells to take on different fates during organ initiation, mediating communication between different tissues during gravitropism and lateral root emergence, and providing long-distance signalling between different plant organs.

Here we focus on the hormone GA, which is a key regulator of plant growth (Achard et al. 2009; Nelissen et al. 2012; Ubeda-Tomás et al. 2008, 2009). In particular, within plant roots and monocotyledonous leaves, cells occupy three main developmental zones: cells closest to the root apex or leaf base are located in the meristem, where they undergo division and little growth; as they leave the meristem, the cells enter the elongation zone, where they rapidly increase in length due to growth; and, eventually, cells reach the mature zone, where the cell lengths stop increasing. The sizes of the meristem and elongation zone are key parameters that determine overall organ growth rates (Gázquez and Beemster 2017) and are dynamically controlled by hormone distributions (Moubayidin et al. 2009; Ubeda-Tomás et al. 2012). For example, it has been shown that GA regulates growth, in part, by controlling the size of the meristem (thus controlling the cell production rate) (Ubeda-Tomás et al. 2009; Achard et al. 2009; Nelissen et al. 2012). Thus, understanding which processes affect the GA distribution is key to understanding this growth regulation.

Our focus on GA transport is also partly motivated by the recent discoveries of membrane transporter proteins of the NPF family (molecular structures that aid hormone transport across cellular and subcellular membranes) (Binenbaum et al. 2023; Tal et al. 2016) that transport both GA and ABA. Some of these transporters, such as NPF 2.12, 2.13, and 3.1 are involved in intercellular transport being localised on the cytoplasmic membrane of certain cell types, whereas NPF 2.14 is a novel transporter localised on the tonoplast (vacuolar membrane) and thus involved in intracellular transport. How such transport into the subcellular vacuole compartment affects the GA distribution is an open question.

### Previous Models of Hormone/Morphogen Dynamics

Multicellular modelling has provided substantial insights into the hormone distributions that underlie plant development. Such models have provided insights into how hormone transport regulates developmental zonation (Grieneisen et al. 2007; Di Mambro et al. 2017; Salvi et al. 2020), lateral root initiation (Xuan et al. 2016; van den Berg et al. 2021; Santos Teixeira et al. 2022), gravitropism (Swarup et al. 2005), root hair growth (Jones et al. 2009), halotropism (van den Berg et al. 2016), root vasculature patterning (Muraro et al. 2014; el Showk et al. 2015), leaf venation (Mitchison et al. 1981; Feugier and Iwasa 2006; Bayer et al. 2009) and phyllotaxis (Smith et al. 2006; Jönsson et al. 2006; Stoma et al. 2008). Such modelling has also provided insights into the roles of specific processes, for example, how auxin regulation of its own transport creates emergent patterns (e.g. Wabnik et al. 2010, Allen and Ptashny 2020).

Similarly, multicellular modelling has been used to study morphogen dynamics and the effect of its spatial distribution in animals. The concept that spatial distributions of morphogens can control developmental patterning was proposed through Wolpert’s “French flag” model (Wolpert 1969) that suggested that changes in cellular positional information underlie pattern regulation enabling organic systems (with applications to sea urchins) to form patterns even when parts of them are removed or added in a size-invariant manner. More recently, models of morphogen gradients have investigated patterning precision in linear and non-linear morphogen degradation (Adelmann et al. 2023), regeneration of the spinal cord in axolotls (Rodrigo Albors et al. 2015; Rost et al. 2016; Cura Costa et al. 2021), and long-range effects of morphogen gradients in zebrafish (White et al. 2007).

Several multicellular models of GA dynamics in files of cells have previously been developed (Band et al. 2012, 2022; Rizza et al. 2021), and have provided insights into how growth-induced dilution (Band et al. 2012) and GA metabolism (Band et al. 2022; Rizza et al. 2021) affect the GA distribution. The initial model (Band et al. 2012) considered cell growth only representing the root elongation zone and the formulation was subsequently developed to include the meristem and cell division (Band et al. 2022; Rizza et al. 2021). While these models incorporated subcellular vacuolar compartments, transport across the tonoplast is assumed to be rapid and passive. Furthermore, these models either do not consider hormone transport between cells (Band et al. 2022; Rizza et al. 2021) or assume this to be a small effect (Band et al. 2012). GA transport was however, previously investigated in a model of the root cross-section (Binenbaum et al. 2023), which revealed how the localization of the NPF transporters leads to accumulation in the endodermal layer, and suggested that the vacuole acts as a storage vessel that can release GA in regions where it is not readily supplied.

### Previous Studies Using Multiscale Asymptotic Analysis

Multiscale asymptotic analysis has been used to investigate numerous biological systems including calcium dynamics (Goel et al. 2006), pattern formation (Newell et al. 2008), nutrient distribution (Shipley et al. 2009), and viral dynamics (Rüdiger et al. 2019). Several studies have applied multiscale asymptotic analysis to models that consider cell growth due to biomechanical processes (Middleton et al. 2014; Murphy et al. 2019; Piatnitski and Ptashnyk 2020; Tambyah et al. 2020). In Tambyah et al. (2020), for example, they present a model for epithelial tissue dynamics with diffusive transport of chemical species between cells. They begin with a discrete coupled mechanobiological model incorporating elastic forces between cells due to growth and derive a continuum macroscale effective reaction–advection–diffusion equation for the species transport.

Relating tissue-scale dynamics to the cell-scale processes has been much exploited in analysing plant growth dynamics, whereby kinematic analysis has been used to relate tissue-scale measurements to the underlying cellular behaviours (e.g. Beemster and Baskin 1998, Silk and Erickson 1979). Far fewer studies have considered such relationships in the context of hormone transport, with the majority of models focusing on computational simulations (as listed in Sect. 1.2 above). Notable examples applying asymptotic analysis to examine the transport of the plant hormone auxin include Mitchison and Brenner (1980), Kramer (2002), Chavarría-Krauser and Ptashnyk (2010), and Band and King (2012). These studies considered how cellular parameters and polar auxin transporters lead to a directed hormone flux, characterised by an auxin velocity. Of particular relevance here, is the work of Mitchison and Brenner (1980) who discuss the role of the subcellular vacuole compartment in reducing the effective auxin velocity. In addition, a continuum approximation for a GA model was developed in Band et al. (2022) (albeit with no cell-to-cell transport) in order to reduce simulation times and enable parameter estimation.

### Paper Layout

In this paper, we aim to further understand the role of transport into the subcellular vacuolar compartments by focussing on the longitudinal direction of the root, and assessing how membrane transport combined with hormone dilution impacts longitudinal hormone transport. We aim to determine how tissue-scale transport depends on the combined effect of both cellular and subcellular (through the vacuole and the continuous apoplast) hormone transport along a single file of growing cells of the same type. Furthermore, unlike previous analysis, where cell growth is linked to other processes, such as elastic forces, here, we will explicitly prescribe the growth and division dynamics, and spatial variance across cell sizes to study their effect on the hormone transport.

The paper is structured as follows. We first focus on the role of subcellular compartments, considering these in a file of non-growing cells in Sect. 2. In Sects. 2.1–2.4, we present the dimensional and non-dimensionalised discrete model for hormone transport, together with the set of dimensional and dimensionless parameters. In Sect. 2.5, we use asymptotic analysis to derive a continuum macroscopic model for the hormone concentration along the file, and consider how the cell geometries and transport processes influence the derived effective diffusivity. In Sect. 2.6, we present numerical results, comparing the discrete and the continuum models and exploring the effect of varying the model parameters. The analysis reveals how the cell-scale parameters contribute to the effective tissue-scale diffusivity, providing insights into the role of the transporters, plasmodesmata, geometry, and pH (Sects. 2.5–2.6). In Sects. 3.1–3.2, we extend the model and analysis to consider a file of growing cells with spatially varying lengths. In Sects. 3.3 and 3.4, we consider two base cases of growing identical cells, and static cells with spatially varying lengths, and, in Sect. 3.5, we discuss the origin of the induced effective velocity. In Sect. 3.6, we present the corresponding numerical results and how they depend on parameter variation. Given growing cells in biological tissues typically divide to maintain viable cell sizes, we conclude our analysis with a short Sect. 4 which describes how cell division affects the hormone transport dynamics. Finally, we discuss biological significance and draw conclusions in Sect. 5.

## Model of Hormone Transport in a File of Non-growing Identical Cells with Subcellular Compartments

### Model Set-up

**Fig. 1:**
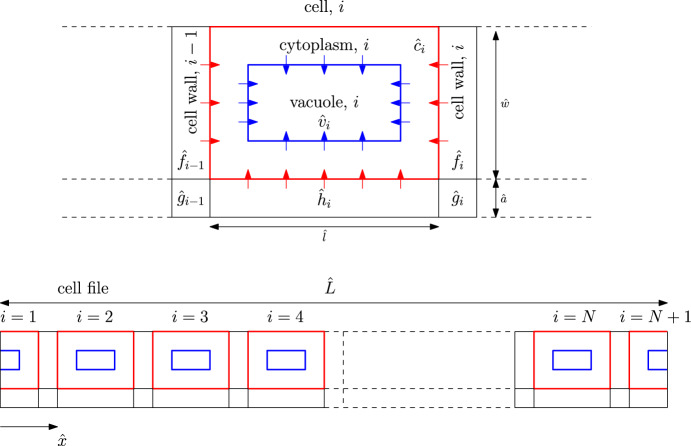
Schematic of a file that consists of repeating cell units. Red arrows represent the cytoplasmic importers (NPF 2.12, 2.13 3.1 in the case of GA), whereas blue arrows represent the vacuolar importer (NPF 2.14 in the case of GA). The cytoplasmic membrane is shown in red, and the tonoplast is shown in blue (Color figure online)

We begin by considering a file of *N* cells with subcellular comparments, considering cells that do not grow and are identical. For simplicity, we consider a two-dimensional geometry. We compartmentalise each cell into rectangular sections for the cytoplasm, cell wall (apoplast), and vacuole (as shown in Fig. 1). We assume that the vacuole is centrally aligned within the cytoplasm and occupies a prescribed area fraction, $\phi$ (Kaiser and Scheuring 2020; Dünser et al. 2022), of the symplastic part of the cell (the cell without the apoplast). Furthermore, even though the whole apoplast is continuously connected, for analytical purposes, we split it into “longitudinal” compartments aligned with the file length, “transverse” compartments between cells, and “corner” compartments that join them together. We let the length and width of the cytoplasm compartment be ${\hat{l}}$ and ${\hat{w}}$, respectively, and the thickness of the cell-wall compartments be ${\hat{a}}$, so that the total length of the cell file is calculated to be ${\hat{L}}=N({\hat{l}}+{\hat{a}})$. We assume that the vacuole dimensions are in the same proportions as the dimensions of the cell (i.e. the vacuoles have length $\sqrt{\phi }{\hat{l}}$ and width $\sqrt{\phi }{\hat{w}}$), and thus the length of the tonoplast (membrane of the vacuole) is given by $2 \sqrt{\phi } ({\hat{l}}+{\hat{w}})$. We note that the assumed rectangular shape of the vacuole is an idealised, yet not unrealistic, representation of a real cell, especially when the vacuole is large (Kramer and Ackelsberg 2015; Kaiser and Scheuring 2020). The focus here is on developing the mathematical tools necessary to model such a system, but, in principle, any shape of the vacuole can be appropriately incorporated into the model. We also denote the hormone concentration within each of the cytoplasm, vacuole, and the “transverse,” “corner,” and “longitudinal” cell wall compartments by ${\hat{c}}_{i}, {\hat{v}}_{i}, {\hat{f}}_{i}, {\hat{g}}_{i}$, and ${\hat{h}}_{i}$, respectively, where the index *i* ($1 \le i \le N$) refers to the *i*-th cell in the file (see Fig. 1 for a schematic of the cell-file geometry). We note that the chosen representation of the repeating cell unit is equivalent to considering a symmetric cell with horizontal apoplastic compartments (of half their original thickness) on either side of it, as explained in Appendix A. Finally, we assume that the cell file is isolated from its surroundings and there is no flux of hormone across its external boundary.

Hormones, such as GA, ABA, and auxin, can exist in both protonated and anionic form within the cell, where only the protonated form can passively diffuse through the cell membrane and the tonoplast down a concentration gradient. The anionic form is transported across them down an electrochemical gradient via protein transporters that can either be importers or exporters, depending on whether they carry the hormone into or out of the compartment. The ratio of protonated to anionic hormone within the cell is determined by the pH and the equilibrium dissociation constant, pK. In particular, the proportions of protonated hormone in the cell apoplast, cytoplasm, and vacuole, respectively, are given by

1 $$\begin{aligned} A_{1}=\frac{1}{1+10^{\mathrm {pH_{apo}}-\textrm{pK}}}, \qquad B_{1}=\frac{1}{1+10^{\mathrm {pH_{cyt}}-\textrm{pK}}}, \qquad C_{1}=\frac{1}{1+10^{\mathrm {pH_{vac}}-\textrm{pK}}}, \end{aligned}$$

where $\mathrm {pH_{apo}}$, $\mathrm {pH_{cyt}}$, and $\mathrm {pH_{vac}}$ are the apoplastic, cytoplasmic, and vacuolar pH, respectively. Thus, for example, the protonated hormone concentration in the cytoplasm of cell *i* is given by $A_{1}{\hat{c}}_{i}$, whereas $(1-A_{1}){\hat{c}}_{i}$ denotes the corresponding anionic hormone concentration. The passive flux from the cytoplasm into the “longitudinal” apoplastic compartment of cell *i* is then given by

2 $$\begin{aligned} {\hat{P}}_{\textrm{pass}}(B_{1}{\hat{c}}_{i}-A_{1}{\hat{h}}_{i}), \end{aligned}$$

where ${\hat{P}}_{\textrm{pass}}$ is the passive membrane permeability. The low pH in the apoplast and vacuoles results in a substantial proportion of the hormone being protonated and able to passively diffuse across the membranes, whereas in the cytoplasm, where the pH is around 7, very little hormone is protonated, and nearly all requires transporters to facilitate membrane transport (see Table 1 for the values relevant to GA).

Transport of plant hormones across cell membranes via protein transporters depends on the localisation and nature of the membrane protein in a particular tissue, with different membrane proteins transporting different hormones. In this study, we focus on the specific case of GA transport, although by redefining the parameter groupings introduced in (9) below, the analysis can be easily adapted to any other hormone following a similar transport mechanism.

Motivated by the recent scientific discoveries of NPF transporters for GA and ABA (Binenbaum et al. 2023; Tal et al. 2016), namely, NPF 2.12, 2.13, 2.14, and 3.1, we assume we have an importer localised on the cytoplasmic membranes transporting hormone out of the apoplast into the cytoplasm (*cf.* NPF 2.12, 2.13, 3.1), and an exporter localised on the tonoplast transporting hormone out of the cytoplasm into the vacuole (thus, also called a vacuolar importer, *cf.* NPF 2.14).

In order to describe the flux associated with the facilitated transport via these transporters, we follow previous plant hormone models using the Goldman–Hodgkin–Katz theory (Band et al. 2014; Band and King 2012; Mellor et al. 2020). As explained in Band and King (2012), the flux from the cytoplasm into the “longitudinal” apoplastic compartment, for example, is given by

3 $$\begin{aligned} {\hat{P}}_{\textrm{imp}}(B_{2}{\hat{c}}_{i}-A_{2}{\hat{h}}_{i}), \end{aligned}$$

where ${\hat{P}}_{\textrm{imp}}$ is the permeability of the importer, and $A_{2}$ and $B_{2}$ are given by

4 $$\begin{aligned} A_{2}=q(\tilde{\phi }_{\textrm{mem}})(1-A_{1}), \qquad B_{2}=q(-\tilde{\phi }_{\textrm{mem}})(1-B_{1}), \end{aligned}$$

with

5 $$\begin{aligned} q(\tilde{\phi }_{\textrm{mem}})=\frac{\tilde{\phi }_{\textrm{mem}}}{\textrm{e}^{\tilde{\phi }_{\textrm{mem}}}-1}, \qquad \tilde{\phi }_{\textrm{mem}}=\frac{F_{D} {\hat{V}}_{\textrm{mem}}}{R {\hat{T}}}, \end{aligned}$$

where $F_{D}$ is Faraday’s constant, ${\hat{V}}_{\textrm{mem}}$ is the potential difference across the cytoplasmic membrane, *R* is the universal gas constant, and ${\hat{T}}$ is the absolute temperature. Similarly, the flux from the cytoplasm into the vacuole is given by

6 $$\begin{aligned} {\hat{P}}_{\textrm{exp}}(B_{3}{\hat{c}}_{i}-C_{3}{\hat{v}}_{i}), \end{aligned}$$

where ${\hat{P}}_{\textrm{exp}}$ is the permeability of the exporter, and $B_{3}$ and $C_{3}$ are given by

7 $$\begin{aligned} B_{3}=q(\tilde{\phi }_{\textrm{ton}})(1-B_{1}), \qquad C_{3}=q(-\tilde{\phi }_{\textrm{ton}})(1-C_{1}), \end{aligned}$$

with

8 $$\begin{aligned} \tilde{\phi }_{\textrm{ton}}=\frac{F_{D} {\hat{V}}_{\textrm{ton}}}{R {\hat{T}}}, \end{aligned}$$

where ${\hat{V}}_{\textrm{ton}}$ is the potential difference across the tonoplast.

For convenience, we define the parameter groupings

9 $$\begin{aligned} {\hat{P}}_{\textrm{ca}}= & {} B_{1} {\hat{P}}_{\textrm{pass}}+B_{2} {\hat{P}}_{\textrm{imp}}, \quad {\hat{P}}_{\textrm{cv}}=B_{1} {\hat{P}}_{\textrm{pass}}+B_{3} {\hat{P}}_{\textrm{exp}},\nonumber \\ {\hat{P}}_{\textrm{ac}}= & {} A_{1} {\hat{P}}_{\textrm{pass}}+A_{2} {\hat{P}}_{\textrm{imp}}, \quad {\hat{P}}_{\textrm{vc}}=C_{1} {\hat{P}}_{\textrm{pass}}+C_{3} {\hat{P}}_{\textrm{exp}}, \end{aligned}$$

where ${\hat{P}}_{\textrm{ca}}, {\hat{P}}_{\textrm{cv}}, {\hat{P}}_{\textrm{ac}}$, and ${\hat{P}}_{\textrm{vc}}$ are effective permeabilities for hormone transport from the cytoplasm into the apoplast and the vacuole, and from the apoplast and the vacuole into the cytoplasm, respectively.

In addition to transport across cell membranes, plant hormones also diffuse directly between adjacent cytoplasms through plasmodesmata (membrane-lined channels within the cell apoplast) (Rutschow et al. 2011). The associated flux is given by

10 $$\begin{aligned} {\hat{P}}_{\textrm{plas}}({\hat{c}}_{i+1}-{\hat{c}}_{i}), \end{aligned}$$

where ${\hat{P}}_{\textrm{plas}}$ is the permeability associated with transport through the plasmodesmata.

We can now present the total fluxes, ${\hat{J}}_{xyz}$, from compartment *x* into compartment *y* of cell *z* for a general hormone, given by

11a $$\begin{aligned} {\hat{J}}_{cfi}&= {\hat{P}}_{\textrm{ca}} {\hat{c}}_{i} - {\hat{P}}_{\textrm{ac}} {\hat{f}}_{i}, \end{aligned}$$

11b $$\begin{aligned} {\hat{J}}_{cvi}&= {\hat{P}}_{\textrm{cv}} {\hat{c}}_{i} - {\hat{P}}_{\textrm{vc}} {\hat{v}}_{i}, \end{aligned}$$

11c $$\begin{aligned} {\hat{J}}_{fci}&= {\hat{P}}_{\textrm{ac}} {\hat{f}}_{i-1} - {\hat{P}}_{\textrm{ca}} {\hat{c}}_{i}, \end{aligned}$$

11d $$\begin{aligned} {\hat{J}}_{chi}&= {\hat{P}}_{\textrm{ca}} {\hat{c}}_{i} - {\hat{P}}_{\textrm{ac}} {\hat{h}}_{i}, \end{aligned}$$

11e $$\begin{aligned} {\hat{J}}_{fgi}&= \frac{2 {\hat{D}}_{\textrm{apo}}}{{\hat{w}} + {\hat{a}}} ({\hat{f}}_{i}-{\hat{g}}_{i}), \end{aligned}$$

11f $$\begin{aligned} {\hat{J}}_{ghi}&= \frac{2 {\hat{D}}_{\textrm{apo}}}{{\hat{l}} + {\hat{a}}} ({\hat{g}}_{i-1}-{\hat{h}}_{i}), \end{aligned}$$

11g $$\begin{aligned} {\hat{J}}_{hgi}&= \frac{2 {\hat{D}}_{\textrm{apo}}}{{\hat{l}} + {\hat{a}}} ({\hat{h}}_{i}-{\hat{g}}_{i}), \end{aligned}$$

11h $$\begin{aligned} {\hat{J}}_{cci}&={\hat{P}}_{\textrm{plas}}({\hat{c}}_{i+1}-{\hat{c}}_{i}), \end{aligned}$$

The fluxes (11e)–(11g) are between apoplastic compartments and are due to passive hormone diffusion, with diffusivity $D_{\textrm{apo}}$, over the distance between the centres of the corresponding compartments. The flux (11h) is associated with direct passive transport between cell cytoplasms via the plasmodesmata. We assume that the intracellular hormone diffusion is sufficiently fast that the concentration in each compartment can be treated as spatially uniform (noting that the justification for this assumption described in Band and King (2012) also holds for the parameter values used throughout this paper).

### Parameter Values

Before we proceed to describe the full model, in Table 1, we present the physical parameters with their typical values that pertain to the hormone GA in the model species *Arabidopsis thaliana*. We note that there can be short or long cells, and small or large vacuoles, depending on which zone within the plant they are located in. For example, in the root, the cells in the meristem are short and the vacuoles are small, whereas, in the mature zone, the cells have undergone elongation, and the vacuoles have enlarged. We assume the cell lengths are not large enough that the timescale for cytoplasmic diffusion becomes comparable to the timescales for apoplastic diffusion and membrane transport, enabling us to maintain the assumption of spatially uniform concentrations within each compartment (see Section 2.2). Using the parameters from Table 1 and a cytoplasmic diffusivity of $D_{\textrm{c}}=670 \, \upmu \textrm{m}^2/\textrm{s}$ (Kramer et al. 2007), we calculate that this occurs at lengths that are approximately $1200 \, \upmu$m, which is much longer than most of *Arabidopsis* cells.

**Table 1:** Physical parameter estimates for the hormone GA within *Arabidopsis* root tissues

Parameter	Description	Value	References
${\hat{l}}$	Cell length	$20/200 \, \upmu \textrm{m}$	Band and King (2012)
${\hat{w}}$	Cell width	$10 \, \upmu \textrm{m}$	Band and King (2012)
${\hat{a}}$	Apoplast thickness	$0.5 \, \upmu \textrm{m}$	Band and King (2012)
*N*	Number of cells	20 cells	Band and King (2012)
$\phi$	Vacuolar fraction	0.1–0.91	
${\hat{C}}$	Upstream hormone concentration	$1 \, \upmu \textrm{M}$	
pH$_{\textrm{cyt}}$	Cytoplasmic pH	7	Sze (1985)
pH$_{\textrm{apo}}$	Apoplastic pH	5.3	Band et al. (2014)
pH$_{\textrm{vac}}$	Vacuolar pH	5.5	Mathieu et al. (1989), Sze (1985)
pK	Equilibrium dissociation constant	4.2	Kramer (2006)
${\hat{D}}_{\textrm{apo}}$	Apoplastic diffusivity	$32 \, \mathrm {\upmu m^2/s}$	Kramer et al. (2007)
$\hat{V}_{\textrm{mem}}$	Potential across cell membrane	$-120 \, \textrm{mV}$	Band et al. (2014), Sze (1985)
$\hat{V}_{\textrm{ton}}$	Potential across tonoplast	$-30 \, \textrm{mV}$	Hedrich et al. (2018), Sze (1985)
$\hat{V}$	absolute temperature	$300 \, \textrm{K}$	Band et al. (2014)
${\hat{P}}_{\textrm{pass}}$	Passive permeability	$0.333 \, \upmu \textrm{m}/\textrm{s}$	Binenbaum et al. (2023)
${\hat{P}}_{\textrm{imp}}$	Importer permeability	$0.017 \, \upmu \textrm{m}/\textrm{s}$	Binenbaum et al. (2023)
${\hat{P}}_{\textrm{exp}}$	Exporter permeability	$0.556 \, \upmu \textrm{m}/\textrm{s}$	Binenbaum et al. (2023)
${\hat{P}}_{\textrm{plas}}$	Plasmodesmatal permeability	$0.81 \, \upmu \textrm{m}/\textrm{s}$	Rutschow et al. (2011)
$F_{D}$	Faraday’s constant	$96500 \, \textrm{C}/\textrm{mol}$	Band et al. (2014)
*R*	Universal gas constant	$8.31 \, \textrm{J}/\textrm{mol K}$	Band et al. (2014)
${\hat{P}}_{\textrm{ca}}$	$B_{1} {\hat{P}}_{\textrm{pass}}+B_{2} {\hat{P}}_{\textrm{imp}}$	$0.0013 \, \upmu \textrm{m}/\textrm{s}$	[$\star$]
${\hat{P}}_{\textrm{ac}}$	$A_{1} {\hat{P}}_{\textrm{pass}}+A_{2} {\hat{P}}_{\textrm{imp}}$	$0.098 \, \upmu \textrm{m}/\textrm{s}$	[$\star$]
${\hat{P}}_{\textrm{cv}}$	$B_{1} {\hat{P}}_{\textrm{pass}}+B_{3} {\hat{P}}_{\textrm{exp}}$	$0.94 \, \upmu \textrm{m}/\textrm{s}$	[$\star$]
${\hat{P}}_{\textrm{vc}}$	$C_{1} {\hat{P}}_{\textrm{pass}}+C_{3} {\hat{P}}_{\textrm{exp}}$	$0.29 \, \upmu \textrm{m}/\textrm{s}$	[$\star$]
$A_{1}$	$1/(1+10^{\mathrm {pH_{apo}}-\textrm{pK}})$	0.074	[$\star$]
$A_{2}$	$(F_{D} {\hat{V}}_{\textrm{mem}}/R {\hat{T}})(1-A_{1})/(\textrm{e}^{F_{D} {\hat{V}}_{\textrm{mem}}/R {\hat{T}}}-1)$	4.34	[$\star$]
$B_{1}$	$1/(1+10^{\mathrm {pH_{cyt}}-\textrm{pK}})$	0.0016	[$\star$]
$B_{2}$	$-(F_{D} {\hat{V}}_{\textrm{mem}}/R {\hat{T}})(1-B_{1})/(\textrm{e}^{-F_{D} {\hat{V}}_{\textrm{mem}}/R {\hat{T}}}-1)$	0.045	[$\star$]
$B_{3}$	$(F_{D} {\hat{V}}_{\textrm{ton}}/R {\hat{T}})(1-B_{1})/(\textrm{e}^{F_{D} {\hat{V}}_{\textrm{ton}}/R {\hat{T}}}-1)$	1.69	[$\star$]
$C_{1}$	$1/(1+10^{\mathrm {pH_{vac}}-\textrm{pK}})$	0.048	[$\star$]
$C_{3}$	$-(F_{D} {\hat{V}}_{\textrm{ton}}/R {\hat{T}})(1-C_{1})/(\textrm{e}^{-F_{D} {\hat{V}}_{\textrm{ton}}/R {\hat{T}}}-1)$	0.50	[$\star$]

Parameters referenced by [$\star$] are calculated using (1), (4), (5), (7), (8), and (9). The permeability estimates are obtained from oocyte experiments for GA$_{4}$ in our previous work (Binenbaum et al. 2023). Here, for concreteness, we have taken ${\hat{P}}_{\textrm{imp}}$ to refer to the permeability of NPF 2.12, but the corresponding permeability of NPF 3.1 can also be used, and ${\hat{P}}_{\textrm{exp}}$ refers to the permeability of NPF 2.14

### Governing Equations

In order to derive the full discrete multicellular formulation of the model for hormone transport within the file of cells, we relate the rate of change of hormone concentration in each of the compartments to the corresponding net flux of the hormone into that compartment over the area of the corresponding boundary, which is due to both passive and facilitated transport of the hormone across all internal boundaries between compartments. We assume that, at the upstream end of the file, the hormone concentration in the cytoplasm is given by an external value, ${\hat{C}}$, whereas, at the downstream end, we assume a passive boundary condition of no flux of the hormone. We note that, to close the system, we need to prescribe an additional boundary condition at the upstream end associated with the hormone concentration in the corresponding “longitudinal” apoplastic compartment. However, as we will see in the next section, in the physically relevant parameter regime, the hormone concentrations evolve quasi-statically, and there is a dependence of the concentration in the “longitudinal” apoplastic compartments on the cytoplasmic concentration, which will effectively prescribe the boundary condition we need. Initially, we assume that there is no hormone in the cells. We thus obtain the following system of ordinary differential equations in time, ${\hat{t}}$,

12a $$\begin{aligned} (1-\phi ) {\hat{w}} {\hat{l}}\dfrac{\textrm{d} {\hat{c}}_{i}}{\textrm{d} {\hat{t}}}&= {\hat{w}} ({\hat{J}}_{fci}-{\hat{J}}_{cfi}+{\hat{J}}_{cci}-{\hat{J}}_{cc(i-1)})-{\hat{l}} {\hat{J}}_{chi}-2\sqrt{\phi }({\hat{l}}+{\hat{w}}){\hat{J}}_{cvi}, \end{aligned}$$

12b $$\begin{aligned} \phi {\hat{w}} {\hat{l}} \dfrac{\textrm{d} {\hat{v}}_{i}}{\textrm{d} {\hat{t}}}&= 2\sqrt{\phi }({\hat{l}}+{\hat{w}}){\hat{J}}_{cvi}, \end{aligned}$$

12c $$\begin{aligned} {\hat{a}} {\hat{w}} \dfrac{\textrm{d} {\hat{f}}_{i}}{\textrm{d} {\hat{t}}}&= {\hat{w}} ({\hat{J}}_{cfi}-{\hat{J}}_{fc(i+1)})-{\hat{a}} {\hat{J}}_{fgi}, \end{aligned}$$

12d $$\begin{aligned} {\hat{a}}^2 \dfrac{\textrm{d} {\hat{g}}_{i}}{\textrm{d} {\hat{t}}}&= {\hat{a}}({\hat{J}}_{hgi}-{\hat{J}}_{gh(i+1)}+ {\hat{J}}_{fgi}), \end{aligned}$$

12e $$\begin{aligned} {\hat{a}} {\hat{l}} \dfrac{\textrm{d} {\hat{h}}_{i}}{\textrm{d} {\hat{t}}}&= {\hat{l}} {\hat{J}}_{chi} + {\hat{a}} ({\hat{J}}_{ghi}-{\hat{J}}_{hgi}), \end{aligned}$$

which hold for $2 \le i \le N$. This is subject to the following boundary conditions

13a $$\begin{aligned} {\hat{c}}_{1}&= {\hat{C}}, \qquad {\hat{h}}_{1}={\hat{H}}, \end{aligned}$$

13b $$\begin{aligned} {\hat{c}}_{N+1}&= {\hat{c}}_{N-1}, \qquad {\hat{h}}_{N+1} = {\hat{h}}_{N-1}, \end{aligned}$$

and initial conditions

14 $$\begin{aligned} {\hat{c}}_{i}={\hat{v}}_{i}={\hat{f}}_{i}={\hat{g}}_{i}={\hat{h}}_{i}=0 \qquad \text { at } \qquad {\hat{t}}=0 \qquad \text { for } 2 \le i \le N, \end{aligned}$$

where ${\hat{H}}$ is a constant that will be determined in Sect. 2.5.1 from the compatibility conditions between the leading-order contributions of the cytoplasmic and apoplastic concentration.

We note that we have used the formulation in (13b) to express the no-flux condition, following the approach in Band et al. (2012), but the solutions are approximately the same for sufficiently many cells, if we use an alternative formulation, such as ${\hat{c}}_{N+1}={\hat{c}}_{N}, {\hat{h}}_{N+1}={\hat{h}}_{N}$.

### Non-dimensionalisation

We non-dimensionalise the system (12), (13), and (14) using the following scales

15 $$\begin{aligned} {\hat{t}}=({\hat{L}}^2/{\hat{D}}_{\textrm{apo}})t, \quad ({\hat{c}}_{i},{\hat{v}}_{i},{\hat{f}}_{i},{\hat{g}}_{i},{\hat{h}}_{i})={\hat{C}}(c_{i}, v_{i}, f_{i}, g_{i}, h_{i}), \quad {\hat{J}}=\frac{{\hat{D}}_{\textrm{apo}} {\hat{C}}}{{\hat{l}}+{\hat{a}}}J, \nonumber \\ \end{aligned}$$

where we choose to non-dimensionalise time with the timescale associated with diffusion of the hormone through the apoplast across the length of the whole file, since ${\hat{D}}_{\textrm{apo}}$ has been accurately measured (Kramer 2006) and is a parameter that we will not vary. We non-dimensionalise the fluxes using the typical scale for the diffusive apoplastic flux along the cell length, motivated by formula (11e–11g).

From (12), the dimensionless equations therefore read

16a $$\begin{aligned} \frac{(1-\phi ) \epsilon ^2 \omega }{1+\lambda } \dfrac{\textrm{d} c_{i}}{\textrm{d} t}&= \omega (J_{fci}-J_{cfi}+J_{cci}-J_{cc(i-1)})-J_{chi}-2\sqrt{\phi }(1 + \omega )J_{cvi}, \end{aligned}$$

16b $$\begin{aligned} \frac{\phi \epsilon ^2 \omega }{1+\lambda } \dfrac{\textrm{d} v_{i}}{\textrm{d} t}&= 2\sqrt{\phi }(1+\omega )J_{cvi}, \end{aligned}$$

16c $$\begin{aligned} \frac{\epsilon ^2 \lambda \omega }{1+\lambda } \dfrac{\textrm{d} f_{i}}{\textrm{d} t}&= \omega (J_{cfi}-J_{fc(i+1)})-\lambda J_{fgi}, \end{aligned}$$

16d $$\begin{aligned} \frac{\epsilon ^2 \lambda ^2}{1+\lambda } \dfrac{\textrm{d} g_{i}}{\textrm{d} t}&= \lambda (J_{hgi}-J_{gh(i+1)}+ J_{fgi}), \end{aligned}$$

16e $$\begin{aligned} \frac{\epsilon ^2 \lambda }{1+\lambda } \dfrac{\textrm{d} h_{i}}{\textrm{d} t}&= J_{chi} + \lambda (J_{ghi}-J_{hgi}), \end{aligned}$$

where

17a $$\begin{aligned} J_{cfi}&= P_{\textrm{ca}} c_{i} - P_{\textrm{ac}} f_{i}, \end{aligned}$$

17b $$\begin{aligned} J_{cvi}&= P_{\textrm{cv}} c_{i} - P_{\textrm{vc}} v_{i}, \end{aligned}$$

17c $$\begin{aligned} J_{fci}&= P_{\textrm{ac}} f_{i-1} - P_{\textrm{ca}} c_{i}, \end{aligned}$$

17d $$\begin{aligned} J_{chi}&= P_{\textrm{ca}} c_{i} - P_{\textrm{ac}} h_{i}, \end{aligned}$$

17e $$\begin{aligned} J_{fgi}&= \frac{2 (1+\lambda )}{\omega +\lambda } (f_{i}-g_{i}), \end{aligned}$$

17f $$\begin{aligned} J_{ghi}&= 2 (g_{i-1}-h_{i}), \end{aligned}$$

17g $$\begin{aligned} J_{hgi}&= 2 (h_{i}-g_{i}), \end{aligned}$$

17h $$\begin{aligned} J_{cci}&=P_{\textrm{plas}}(c_{i+1}-c_{i}), \end{aligned}$$

with

18 $$\begin{aligned} P_{\text {ac}}=&{} A_{1} P_{\text {pass}}+A_{2} P_{\text {imp}}, \quad P_{\text {ca}}= B_{1} P_{\text {pass}}+B_{2} P_{\text {imp}}, \nonumber \\ P_{\text {cv}}=&{} B_{1} P_{\text {pass}}+B_{3} P_{\text {exp}}, \quad P_{\text {vc}}= C_{1} P_{\text {pass}}+C_{3} P_{\text {exp}}, \end{aligned}$$

in the case of GA transport. Equations (16) and (17) hold for $2 \le i \le N$, except (17c) and (17f), which hold for $2 \le i \le N+1$, and we have defined the following dimensionless parameters

19 $$\begin{aligned} \epsilon =\frac{{\hat{l}}+{\hat{a}}}{{\hat{L}}}=\frac{1}{N},&\quad \lambda =\frac{{\hat{a}}}{{\hat{l}}}, \quad \omega = \frac{{\hat{w}}}{{\hat{l}}}, \nonumber \\ (P_{\text {pass}}, P_{\text {imp}}, P_{\text {exp}},P_{\text {plas}})=&\frac{{\hat{l}}+{\hat{a}}}{{\hat{D}}_{\text {apo}}}({\hat{P}}_{\text {pass}},{\hat{P}}_{\text {imp}},{\hat{P}}_{\text {exp}}, {\hat{P}}_{\text {plas}}). \end{aligned}$$

Here, $\epsilon$ is the ratio of the cell length to the length of the whole file, $\lambda$ is the aspect ratio of the “longitudinal” apoplastic compartments, $\omega$ is the aspect ratio of the cytoplasmic compartments, and $P_{\textrm{pass}}, P_{\textrm{imp}},$ $P_{\textrm{exp}}$, and $P_{\textrm{plas}}$ are the dimensionless passive, importer, exporter, and plasmodesmata permeabilities, non-dimensionalised with the typical apoplastic diffusive rate, which, for a plant cell with average length of $100 \, \upmu \textrm{m}$, as in the elongation zone of the *Arabidopsis* root, is of the same order of magnitude as the dimensional passive permeability. The typical values of these dimensionless parameters relevant to GA transport are presented in Table 2, out of which there are nine independent dimensionless parameters governing the behaviour of our model ($P_{\textrm{pass}}, P_{\textrm{imp}},$ and $P_{\textrm{exp}}$ are absorbed into the definitions of $P_{\textrm{ca}}, P_{\textrm{ac}}, P_{\textrm{cv}},$ and $P_{\textrm{vc}}$). We assume that the cytoplasm area is the same in the short and long cells (given root cells elongate through vacuolar expansion (Dolan and Davies (2004)), thus, with $\phi =0.1$ for short cells with ${\hat{l}}=20 \, \upmu \textrm{m}$ (as appropriate for meristem cells), we set $\phi =0.91$ for long cells with ${\hat{l}}=200 \, \upmu \textrm{m}$ (as appropriate for mature cells).

**Table 2:** Dimensionless parameter estimates, calculated from the parameter estimates in Table 1 using (18) and (19)

Parameter	Value (short/long cells)	Description
$\epsilon$	0.05	Ratio of cell length to file length
$\lambda$	0.025/0.0025	Ratio of apoplast thickness to cell length
$\omega$	0.5/0.05	Ratio of cell width to cell length
$\phi$	0.1/0.91	Vacuolar fraction
$P_{\textrm{pass}}$	0.21/2.09	Dimensionless passive permeability
$P_{\textrm{imp}}$	0.011/0.11	Dimensionless cytoplasmic transporter permeability
$P_{\textrm{exp}}$	0.36/3.48	Dimensionless tonoplast transporter permeability
$P_{\textrm{plas}}$	0.52/5.08	Dimensionless plasmodesmata permeability
$P_{\textrm{ca}}$	0.00083/0.0081	Effective dimensionless permeability
		from cytoplasm to apoplast
$P_{\textrm{ac}}$	0.063/0.62	Effective dimensionless permeability
		from apoplast to cytoplasm
$P_{\textrm{cv}}$	0.60/5.89	Effective dimensionless permeability
		from cytoplasm to vacuole
$P_{\textrm{vc}}$	0.19/1.84	Effective dimensionless permeability
		from vacuole to cytoplasm

The governing equations (16) are subject to the following boundary conditions

20a $$\begin{aligned} c_{1}&= 1, \qquad h_{1}=\Upsilon , \end{aligned}$$

20b $$\begin{aligned} c_{N+1}&= c_{N-1}, \qquad h_{N+1}=h_{N-1}, \end{aligned}$$

and initial conditions

21 $$\begin{aligned} c_{i}=v_{i}=f_{i}=g_{i}=h_{i}=0 \qquad \text { at } \qquad t=0 \qquad \text { for } 2\le i \le N, \end{aligned}$$

where $$\Upsilon ={\hat{H}}/{\hat{C}}$$.

### Model Analysis

We now exploit the size of the dimensionless parameters (19) to systematically reduce the discrete model (16), (17), and (20), which consists of $5 N -2$ equations, to a single continuum equation that describes the GA transport across the tissue. In particular, we use the fact that $\epsilon \ll 1$, i.e., the length of a single cell is much smaller than the length of the cell file. We let ${\hat{x}}={\hat{L}}x$ measure the dimensional continuum distance along the file and be non-dimensionalised with the file length. We assume that one end of the file ($x=0$) is located at the centre of the first cell, and the other end ($x=1$) is located at the centre of the $(N+1)$-st cell, as shown in Fig. 1. We proceed by explicitly converting the discrete concentration variables into continuum variables that have both spatial and temporal dependence using Taylor’s approximation, as in

22 $$\begin{aligned} c_{i+1}(t)=c(x+\epsilon ,t)=c(x,t)+\epsilon \dfrac{\partial c}{\partial x}+\frac{\epsilon ^2}{2}\dfrac{\partial ^2 c}{\partial x^2} + O(\epsilon ^3) \end{aligned}$$

for $\epsilon \ll 1$. We then asymptotically expand all concentration variables in powers of $\epsilon$, as in

23 $$\begin{aligned} c = c^{(0)} + \epsilon c^{(1)} + \epsilon ^2 c^{(2)} + O(\epsilon ^3) \end{aligned}$$

for $\epsilon \ll 1$, where superscripts refer to the asymptotic orders in the expansion of the continuous concentration variables.

#### Leading-Order Continuum Approximation with $\varvec{O(1)}$ Parameters

We begin by considering the richest asymptotic limit when all dimensionless parameters are of *O*(1) magnitude. Noting that the left-hand sides of (16) are $O(\epsilon ^2)$ and substituting for the fluxes from (17), the leading-order governing equations are

24a $$\begin{aligned} 0&= 2\omega (P_{\textrm{ac}}f^{(0)}-P_{\textrm{ca}}c^{(0)}) -P_{\textrm{ca}}c^{(0)}+P_{\textrm{ac}}h^{(0)} -2\sqrt{\phi }(1+\omega )(P_{\textrm{cv}}c^{(0)}-P_{\textrm{vc}}v^{(0)}), \end{aligned}$$

24b $$\begin{aligned} 0&= 2\sqrt{\phi }(1+\omega )(P_{\textrm{cv}}c^{(0)}-P_{\textrm{vc}}v^{(0)}), \end{aligned}$$

24c $$\begin{aligned} 0&= 2\omega (P_{\textrm{ca}} c^{(0)}- P_{\textrm{ac}}f^{(0)}) - \frac{2 \lambda (1+\lambda )}{\omega +\lambda } (f^{(0)}-g^{(0)}), \end{aligned}$$

24d $$\begin{aligned} 0&= 4 \lambda (h^{(0)}-g^{(0)}) + \frac{2 \lambda (1+\lambda )}{\omega +\lambda } (f^{(0)}-g^{(0)}), \end{aligned}$$

24e $$\begin{aligned} 0&= P_{\textrm{ca}}c^{(0)}-P_{\textrm{ac}}h^{(0)} + 4\lambda (g^{(0)}-h^{(0)}). \end{aligned}$$

Thus, the leading-order hormone concentrations form an algebraic system. Equations (24a)–(24e) are linearly dependent, since summing all of them gives zero due to conservation of the hormone. Thus, solving this homogeneous system of linear equations for the leading-order variables, we obtain

25 $$\begin{aligned} f^{(0)}=g^{(0)}=h^{(0)}={\mathcal {P}}_{\textrm{a}} c^{(0)}, \qquad v^{(0)}={\mathcal {P}}_{\textrm{v}} c^{(0)}, \end{aligned}$$

where we have defined

26 $$\begin{aligned} {\mathcal {P}}_{\textrm{a}}=P_{\textrm{ca}}/P_{\textrm{ac}} \approx 0.013, \qquad {\mathcal {P}}_{\textrm{v}}=P_{\textrm{cv}}/P_{\textrm{vc}} \approx 3.20, \end{aligned}$$

which describe the relative hormone transport into and out of the apoplast and the vacuole, respectively. We note that, for consistency, this relationship (25) must hold at the two ends of the file, $x=0$ and $x=1$. Thus, as described in Sect. 2.3, specifying $c^{(0)}=1$ at $x=0$ (from (20a)), determines the necessary boundary condition for the “longitudinal” apoplastic compartment, namely, $h^{(0)}={\mathcal {P}}_{\textrm{a}}$. Thus, in the corresponding boundary condition (20a) for the discrete model, $\Upsilon ={\mathcal {P}}_{\textrm{a}}$, and so ${\hat{H}}={\mathcal {P}}_{\textrm{a}}{\hat{C}}$ in (13).

We note that the ratios in (26) are independent of the cell length, as they are solely a function of the permeability of the corresponding membrane. Thus, with *O*(1) parameters, the apoplastic and vacuolar concentrations are proportional to the cytoplasmic concentration, with constants of proportionality depending on the effective transport permeabilities between the corresponding compartments. Using parameter values relevant for GA (given in Table 2), Eq. (25) suggest that the apoplastic GA concentration at a given cell and time is approximately 0.013 times the cytoplasmic concentration, whereas the vacuolar concentration is approximately 3.2 times the cytoplasmic concentration, which is consistent with the experimental observations in Shani et al. (2013) for vacuolar and nuclear GA concentrations.

In deriving the continuum model, we will present the results in terms of one macroscopic variable, namely, the cytoplasmic concentration, $c^{(0)}(x,t)$. In order to close the system and obtain a governing equation that describes how $c^{(0)}$ varies, we sum the original dimensionless Eqs. (16a)–(16e). This way, we do not have to consider balance at every single order to close the system but rather use the simultaneous cancellations of terms when we do the summation. To $O(\epsilon ^2)$, we obtain

27 $$\begin{aligned}{} & {} \frac{\epsilon ^2 }{1+\lambda } \dfrac{\partial }{\partial t}\left( (1-\phi ) \omega c^{(0)} + \phi \omega v^{(0)} + \lambda \omega f^{(0)} + \lambda ^2 g^{(0)} + \lambda h^{(0)}\right) \nonumber \\{} & {} \quad = \epsilon \left( \omega \left( -P_{\textrm{ac}} \dfrac{\partial f^{(0)}}{\partial x}+ P_{\textrm{ca}} \dfrac{\partial c^{(0)}}{\partial x}\right) +2\lambda \left( -\dfrac{\partial g^{(0)}}{\partial x} + \dfrac{\partial h^{(0)}}{\partial x}\right) \right) \nonumber \\{} & {} \qquad + \frac{\epsilon ^2}{2} \left( \omega \left( P_{\textrm{ac}} \dfrac{\partial ^2 f^{(0)}}{\partial x^2}+ P_{\textrm{ca}}\dfrac{\partial ^2 c^{(0)}}{\partial x^2} +2P_{\textrm{plas}} \dfrac{\partial ^2 c^{(0)}}{\partial x^2} \right) +2\lambda \left( \dfrac{\partial ^2 g^{(0)}}{\partial x^2} + \dfrac{\partial ^2 h^{(0)}}{\partial x^2}\right) \right) \nonumber \\{} & {} \qquad + \epsilon ^2 \left( \omega \left( -P_{\textrm{ac}} \dfrac{\partial f^{(1)}}{\partial x}+ P_{\textrm{ca}}\dfrac{\partial c^{(1)}}{\partial x}\right) +2\lambda \left( -\dfrac{\partial g^{(1)}}{\partial x} + \dfrac{\partial h^{(1)}}{\partial x}\right) \right) . \end{aligned}$$

We note that, from (25), the $O(\epsilon )$-term on the right-hand side of (27) vanishes. In order to evaluate the $O(\epsilon ^2)$-term on the right-hand side of (27), we sum (16a), (16b), and (16e), and consider the result to $O(\epsilon )$. This yields

28 $$\begin{aligned} 0=2 \omega \left( P_{\textrm{ac}} f^{(1)}- P_{\textrm{ca}} c^{(1)}\right) +4\lambda \left( g^{(1)} - h^{(1)}\right) - \omega P_{\textrm{ac}} \dfrac{\partial f^{(0)}}{\partial x} - 2\lambda \dfrac{\partial g^{(0)}}{\partial x}. \end{aligned}$$

Differentiating (28) in *x* and substituting into (27) gives

29 $$\begin{aligned}&\frac{\epsilon ^2 }{1+\lambda } \dfrac{\partial }{\partial t}\left( (1-\phi ) \omega c^{(0)} + \phi \omega v^{(0)} + \lambda \omega f^{(0)} + \lambda ^2 g^{(0)} + \lambda h^{(0)}\right) \nonumber \\&=\frac{\epsilon ^2}{2} \left( \omega P_{\textrm{ca}} \dfrac{\partial ^2 c^{(0)}}{\partial x^2} + 2 \omega P_{\textrm{plas}} \dfrac{\partial ^2 c^{(0)}}{\partial x^2} +2\lambda \dfrac{\partial ^2 h^{(0)}}{\partial x^2} \right) . \end{aligned}$$

Using (25), we can express $v^{(0)}$, $f^{(0)}$, $g^{(0)}$, and $h^{(0)}$ in terms of $c^{(0)}$, and, thus, obtain the following effective diffusion equation for the macroscopic cytoplasmic concentration, which we denote by $C=c^{(0)}$ to distinguish it from its discrete counterpart,

30 $$\begin{aligned} \dfrac{\partial C}{\partial t} = D_{\textrm{eff}} \dfrac{\partial ^2 C}{\partial x^2}, \end{aligned}$$

where the *effective diffusivity* $D_{\textrm{eff}}$ is given by

31 $$\begin{aligned} D_{\textrm{eff}}=\frac{(1+\lambda ) (\omega P_{\textrm{ca}}/2 + \omega P_{\textrm{plas}} + \lambda {\mathcal {P}}_{\textrm{a}})}{(1-\phi ) \omega +\phi \omega {\mathcal {P}}_{\textrm{v}} + \lambda (1+ \omega + \lambda ) {\mathcal {P}}_{\textrm{a}}}. \end{aligned}$$

We note that the first term in the second bracket of the numerator comes from transport across the cell membranes, the second term is associated with plasmodesmatal diffusion, and the third term comes from apoplastic diffusion. To close the continuum model, we prescribe the concentration at $x=0$ and impose no flux at $x=1$, i.e.,

32a $$\begin{aligned} C&=1 \quad \text { at } \quad x=0, \end{aligned}$$

32b $$\begin{aligned} \dfrac{\partial C}{\partial x}&=0 \quad \text { at } \quad x=1. \end{aligned}$$

Initially, there is no hormone present, so we have

33 $$\begin{aligned} C=0 \qquad \text { at } \qquad t=0. \end{aligned}$$

Equations (30)–(33) can be solved explicitly using separation of variables to obtain

34 $$\begin{aligned} C(x,t)= 1-\frac{4}{\pi } \sum _{k=0}^{\infty }\frac{1}{2k+1}\sin {\left( \frac{(2k+1) \pi x}{2}\right) }\exp {\left( -D_{\textrm{eff}}(2k+1)^2 \pi ^2 t/4\right) }. \nonumber \\ \end{aligned}$$

We now consider the physically relevant limits and other cases of mathematical interest that can be distilled as sub-limits of this limit.

#### Physically Relevant Limit for Short Cells

Based on the parameter values listed in Tables 1 and 2, we consider the physically relevant limit for GA transport in short cells with ${\mathcal {P}}_{\textrm{a}}, \lambda = O(\epsilon )$ and $P_{\textrm{ca}}=O(\epsilon ^2)$. We, thus, write $({\mathcal {P}}_{\textrm{a}}, \lambda )= \epsilon (\tilde{\mathcal {P}}_{\textrm{a}}, \tilde{\lambda })$ and $P_{\textrm{ca}}=\epsilon ^2 \bar{P}_{\textrm{ca}}$ with $\mathcal {\tilde{P}}_{\textrm{a}}, \bar{P}_{\textrm{ca}}, \tilde{\lambda } = O(1)$. This means that the rate of GA transport across the cytoplasmic membrane is slower than the rate across the tonoplast, and, furthermore, transport from the apoplast into the cytoplasm dominates over transport from the cytoplasm into the apoplast. Considering the balances in (25), this implies the apoplastic concentration is an order of magnitude smaller than the cytoplasmic concentration, i.e., $f^{(0)}, g^{(0)}, h^{(0)} = O(\epsilon )$.

We first describe the continuum limit with no GA transport through the plasmodesmata ($P_{\textrm{plas}}=0$). In this case, the evolution of the cytoplasmic concentration is governed by

35a $$\begin{aligned} \dfrac{\partial C}{\partial \bar{t}}= & {} \bar{D}_{\textrm{eff}} \dfrac{\partial ^2 C}{\partial x^2}, \end{aligned}$$

35b $$\begin{aligned} \bar{D}_{\textrm{eff}}= & {} \frac{\omega \bar{P}_{\textrm{ca}}/2 + \tilde{\lambda }\tilde{\mathcal {P}}_{\textrm{a}}}{(1-\phi ) \omega +\phi \omega {\mathcal {P}}_{\textrm{v}}}, \end{aligned}$$

subject to boundary conditions (32) and initial condition $C=0$ at $\bar{t}=0$, where we have rescaled time and the diffusivity as $t=\bar{t}/\epsilon ^2$ and $D_{\textrm{eff}}= \epsilon ^2 \bar{D}_{\textrm{eff}}$, respectively, to balance the time derivative with the slow diffusion in this case.

Using the values in Tables 1 and 2, we see that the dimensional effective diffusivity (taking into account the rescaling in time) is ${\hat{D}}_{\textrm{eff}}=(0.35 \epsilon ^2) {\hat{D}}_{\textrm{apo}} \approx 28 \times 10^{-3} \, \upmu {\mathrm m^2/s}$. Considering the magnitude of the two numerator terms in (35b), we find that $40\%$ of the effective diffusivity occurs cell-to-cell (i.e. through the cell membranes), with the remaining $60\%$ of the diffusivity occurring through the adjacent layer of apoplast.

#### Physically Relevant Limit for Long Cells

We now consider the physically relevant limit for GA transport in long cells, where ${\mathcal {P}}_{\textrm{a}}, \omega = O(\epsilon )$, $\mathcal {P}_{\textrm{ca}}, \lambda =O(\epsilon ^2)$. We write $(\tilde{\mathcal {P}}_{\textrm{a}}, \omega )= \epsilon (\tilde{\mathcal {P}}_{\textrm{a}}, \tilde{\omega })$ and $(P_{\textrm{ca}}, \lambda )=\epsilon ^2 (\bar{P}_{\textrm{ca}}, \bar{\lambda })$ with $\tilde{P}_{\textrm{a}}, \bar{P}_{\textrm{ca}}, \tilde{\omega }, \bar{\lambda } = O(1)$. Thus, the transport properties are the same as above, and the only difference are the aspect ratios of the cell and the “longitudinal” apoplastic compartment.

In this case (with $P_{\textrm{plas}}=0$), we again need to rescale time and the diffusivity as $t=\bar{t}/\epsilon ^2$ and $D_{\textrm{eff}}= \epsilon ^2 \bar{D}_{\textrm{eff}}$, respectively. Thus, (32), (33), and (35a) hold with effective diffusivity

36 $$\begin{aligned} \bar{D}_{\textrm{eff}}=\frac{\tilde{\omega } \bar{P}_{\textrm{ca}} /2 + \bar{\lambda } \tilde{\mathcal {P}}_{\textrm{a}} }{(1-\phi ) \tilde{\omega } +\phi \tilde{\omega } {\mathcal {P}}_{\textrm{v}}}, \end{aligned}$$

which is the same as (35b), except for the rescaled aspect ratios $\tilde{\omega }$ and $\bar{\lambda }$.

Using the value in Table 1, we find that for long cells, the effective diffusivity is larger (${\hat{D}}_{\textrm{eff}}= (0.63 \epsilon ^2) {\hat{D}}_{\textrm{apo}} \approx 50 \times 10^{-3} \, \upmu {\mathrm m}^2/\textrm{s}$), and a higher proportion of the effective diffusivity occurs through the cell-to-cell pathway (from the magnitude of the terms in the numerator of (36), the cell-to-cell component forms $87\%$ of the effective diffusivity for long cells).

#### The Effect of Plasmodesmata

We now consider the effect of plasmodesmatal diffusion. From Table 2, we see that, in both the short-cell and long-cell limits, $P_{\textrm{plas}}=O(1)$. Thus, (30), (32), and (33) hold (with no time rescaling), and the effective diffusivity in both limits is

37 $$\begin{aligned} D_{\textrm{eff}}=\frac{P_{\textrm{plas}}}{1-\phi +\phi {\mathcal {P}}_{\textrm{v}}}. \end{aligned}$$

For short cells, ${\hat{D}}_{\textrm{eff}}= 0.43 {\hat{D}}_{\textrm{apo}} \approx 14 \, \upmu {\mathrm m^2/s}$, whereas, for long cells, ${\hat{D}}_{\textrm{eff}}= 1.7 {\hat{D}}_{\textrm{apo}} \approx 54 \, \upmu {\mathrm m}^2/\textrm{s}$.

Thus, we see that, provided the plasmodesmata are open, transport through them dominates over the passive and facilitated transport across the cell membrane, which do not contribute to leading-order effective diffusion. This is because the derived formulae (35b) and (36) show that the effective diffusivity associated with the cell-membrane transport is proportional to the effective permeability from the cytoplasm into the apoplast, $P_{\textrm{ca}}$. This permeability is small, because the high cytoplasmic pH causes the majority of the hormone (99.84$\%$ in the case of GA) to be anionic which cannot passively diffuse from the cytoplasm into the apoplast. We see a dramatic increase in the effective diffusivity in the presence of open plasmodesmata, as plasmodesmata provide a direct pathway between the cytoplasms of adjacent cells for hormone diffusion. In practice, though, plasmodesmata can be physically restricted by the presence of callose (De Storme and Geelen 2014), which substantially decreases their permeability. From (35b) and (36), we see that the passive and facilitated transport through the cell membrane become non-negligible when $P_{\textrm{plas}}=O(\epsilon ^2)$, which is when all three transport mechanisms contribute at a similar rate.

We note that the transport across the tonoplast is important even when plasmodesmata are open, and decreases the effective diffusivity the larger the vacuoles are, since this increases the uptake of hormone via the tonoplast transporter from the cytoplasm into the vacuole.

#### Effective Diffusivities in Loss-of-Function Mutants

Given that (31) is the *O*(1) effective diffusivity, we can use it to derive the diffusivities in the loss-of-function mutants for GA transport where there is no cytoplasmic importer (NPF 2.12, NPF 2.13, and NPF 3.1), no vacuolar importer (NPF 2.14), or neither.

When there is no cytoplasmic importer, $P_{\textrm{imp}}=0$, and thus $P_{\textrm{ac}}=A_{1} P_{\textrm{pass}}, P_{\textrm{ca}}=B_{1} P_{\textrm{pass}}$, and the effective diffusivity becomes

38 $$\begin{aligned} D_{\textrm{eff}}=\frac{(1+\lambda ) (\omega B_{1} P_{\textrm{pass}}/2 + \omega P_{\textrm{plas}} + \lambda B_{1} /A_{1})}{(1-\phi ) \omega +\phi \omega {\mathcal {P}}_{\textrm{v}} + \lambda (\omega + \lambda + 1) B_{1}/A_{1}}. \end{aligned}$$

When there is no vacuolar importer, $P_{\textrm{exp}}=0$, and thus $P_{\textrm{cv}}=B_{1} P_{\textrm{pass}}, P_{\textrm{vc}}=C_{1} P_{\textrm{pass}}$, and so

39 $$\begin{aligned} D_{\textrm{eff}}=\frac{(1+\lambda ) (\omega P_{\textrm{ca}}/2 + \omega P_{\textrm{plas}} + \lambda {\mathcal {P}}_{\textrm{a}})}{(1-\phi ) \omega +\phi \omega B_{1}/C_{1} + \lambda (\omega + \lambda + 1) {\mathcal {P}}_{\textrm{a}}}. \end{aligned}$$

When there are no cytoplasmic nor vacuolar importers, we have

40 $$\begin{aligned} D_{\textrm{eff}}=\frac{(1+\lambda ) (\omega B_{1} P_{\textrm{pass}}/2 + \omega P_{\textrm{plas}} + \lambda B_{1} /A_{1})}{(1-\phi ) \omega +\phi \omega B_{1}/C_{1} + \lambda (\omega + \lambda + 1) B_{1}/A_{1}}. \end{aligned}$$

Similarly, we obtain the formulae for the effective diffusivity in the cases of short and long cells with or without plasmodesmata for these loss-of-function mutants from (35b), (36), and (37) by taking $P_{\textrm{ca}}=B_{1} P_{\textrm{pass}}$ and ${\mathcal {P}}_{\textrm{a}}=B_{1}/A_{1}$ in the case of no cytoplasmic importer, ${\mathcal {P}}_{\textrm{v}}=B_{1}/C_{1}$ in the case of no vacuolar importer, and all of these when there are no transporters, as above.

In Table 3, we present the values for the dimensionless effective diffusivities in the short-cell and long-cell limits from Sects. 2.5.2–2.5.5, noting that, in the case of no vacuolar importer, ${\mathcal {P}}_{\textrm{v}}=B_{1}/C_{1}=O(\epsilon )$, and, in the case of long cells $(1-\phi ) \omega \sim \phi \omega B_{1}/C_{1}$. We see that the presence of a vacuolar importer significantly slows down diffusion by storing GA into the vacuole, especially in the long-cell limit, when the vacuole takes up almost the entire cellular space. We also note that, for short cells, the transporters are reducing the effective diffusivity, whereas, for long cells, the cytoplasmic importer increases it, since this counteracts the effect of the transport across the tonoplast, which is dominant. Adding plasmodesmata changes the results only quantitatively, except that the presence of a cytoplasmic importer has no effect on the effective diffusivity due to the dominant transport through the plasmodesmata.

**Table 3:** Dimensionless diffusivities (in scaled time $t=\bar{t}/\epsilon ^2$ for the case without plasmodesmata) for wild type and loss-of-function mutants with and without plasmodesmata for short and long cells, calculated using formulae (35b)–(40) in the relevant limit, and parameter estimates in Table 2

	All transporters	No cytoplasmic	No tonoplast	No transporters
		importer	importer	
Without plasmodesmata	0.35/0.63	0.41/0.37	0.48/15.71	0.56/9.14
(short/long cells)				
With plasmodesmata	0.43/1.70	0.43/1.70	0.58/42.18	0.58/42.18
(short/long cells)				

### Numerical Results

**Fig. 2:**
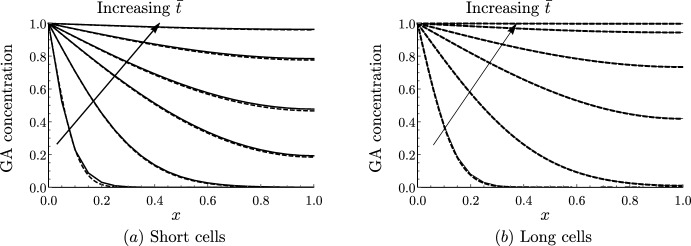
Continuum model predictions agree with discrete model. Plots of the spatial profile of the cytoplasmic GA concentration *c* from the discrete model (solid black) and the macroscopic cytoplasmic GA concentration *C* from the continuum model (dashed black) at $\bar{t}=0.01$, $\bar{t}=0.1$, $\bar{t}=0.5$, $\bar{t}=1$, $\bar{t}=2$, and $\bar{t}=4$ for **a** short and **b** long cells. Parameter values given in Table 2 with $P_{\textrm{plas}}=0$

We first compare simulations of the discrete and continuum models to verify that the continuum representation reproduces the modelled dynamics. We solve the discrete model (16) subject to (20) and (21) using *Mathematica* and compare the results with the solution to the continuum model (30) and (31), subject to (32) and (33), which we solve using the method of lines with 100 grid points in space. For short and long cells, we see excellent agreement between the discrete and the continuum model even for $N=20$ cells (Fig. 2). We note that diffusion is faster in the long cells, and the GA concentration reaches more quickly steady state ($c=C=1$) than in the short cells (consistent with the effective diffusivities calculated in Table 3). This is because, in a file of long cells, for a fixed distance, GA has to cross a cell membrane fewer times than going through short cells. We see that this effect dominates over the opposite effect of large vacuoles in long cells taking up more GA than smaller vacuoles in short cells, which effectively reduces the diffusivity.

**Fig. 3:**
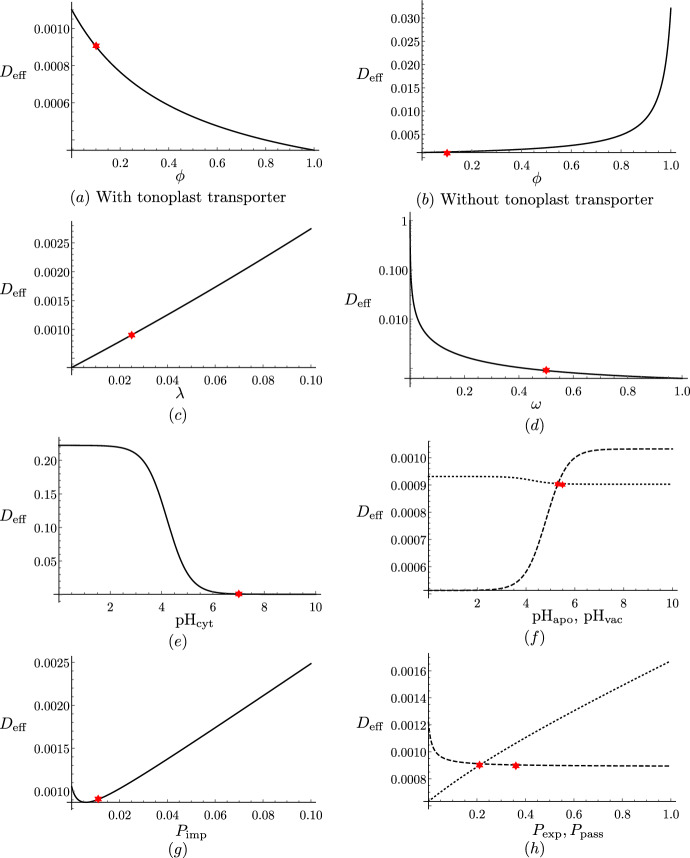
Effect of varying system parameters on effective diffusivity. Plots of the effective diffusivity, $D_{\textrm{eff}}$, from (31) (for short cells with $P_{\textrm{plas}}=0$) as a function of **a** the vacuolar fraction, $\phi$, **b** the vacuolar fraction, $\phi$, without a tonoplast transporter, **c** the dimensionless thickness of the apoplast, $\lambda$, **d** the dimensionless width of the cytoplasm, $\omega$, on a log-plot, **e** the cytoplasmic pH, **f** the apoplastic (dashed) and vacuolar (dotted) pH, **g** the cytoplasmic importer permeability, and **h** the tonoplast exporter (dashed) and passive (dotted) permeability. The red stars indicate the base parameter values corresponding to short cells (see Tables 1 and 2) (Color figure online)

In Fig. 3, we show how varying the model parameters influences the effective diffusivity (31), keeping the remaining parameters at their estimated value (Table 2). We present results for short cells, noting that plots of the effective diffusivity for long cells are qualitatively similar.

We see that, when there is a tonoplast transporter, the effective diffusivity monotonically decreases with the vacuolar proportion (Fig. 3a), since larger vacuoles take up more GA, thereby reducing its effective transport along the file, consistent with the results in Mitchison and Brenner (1980). However, when there is no tonoplast transporter, we observe the opposite behaviour (Fig. 3b). This is because, in this case, there is little transport of GA into the vacuole: ${\mathcal {P}}_{\textrm{v}}=B_{1}/C_{1} \approx 0.03 \ll 1$, and thus the first term in the denominator of (31) dominates over the second term, which dictates the monotonically increasing behaviour. Furthermore, we see that, as the apoplast thickness increases, or the cytoplasm width decreases, the effective diffusivity increases (Fig. 3c and d). This is because either of these changes enlarges the proportion of the apoplast within the tissue width, where GA diffuses more rapidly. We note that, in the extreme cases, as $\lambda \rightarrow \infty$ or $\omega \rightarrow 0$, $D_{\textrm{eff}} \rightarrow 1$, which corresponds to the apoplastic diffusivity.

We observe that decreasing the cytoplasmic pH substantially increases the effective diffusivity (Fig. 3e). This is because, at lower cytoplasmic pH, more of the hormone is in a protonated form, which readily diffuses through the membrane without transporters. In contrast, we see that the values of the apoplastic and vacuolar pH have limited effect on the effective diffusivity (Fig. 3f).

We see that the effective diffusivity increases almost linearly with the cytoplasmic importer permeability, $P_{\textrm{imp}}$, except for very small values of the permeability, where the diffusivity initially decreases (Fig. 3g). This behaviour is due to competition between the cytoplasmic ($\omega P_{\textrm{ca}}/2$) and apoplastic ($\lambda {\mathcal {P}}_{\textrm{a}}$) contributions to the effective diffusion, with the cytoplasmic contribution dominating at larger importer permeabilities. We see that the effective diffusivity monotonically increases with the passive permeability, as expected, whereas it monotonically decreases with the tonoplast exporter permeability, quickly approaching a minimum threshold value (Fig. 3h). This suggests that the precise numerical value for the tonoplast exporter permeability is not significant for the transport dynamics.

For clarity, Fig. 3 has been made with $P_{\textrm{plas}}=0$. With open plasmodesmata, the plasmodesmatal diffusion dominates (as detailed in Sect. 2.5.4), and therefore the effective diffusivity increases with the plasmodesmatal permeability, and the values of importer permeability and apoplastic pH have very little effect. As we can see from the formula (37), parameters that increase the vacuolar concentration relative to the cytoplasmic concentration, ${\mathcal {P}}_{\textrm{v}}$, lead to a decrease in the effective diffusivity, as more hormone is stored in the vacuole.

In summary, this section has reduced a detailed discrete model for hormone transport within a file of cells with subcellular compartments (comprising $5N-2$ ODEs) to a single partial differential equation for the macroscopic cytoplasmic hormone concentration across the file. The derived effective diffusivity provides understanding of precisely how the tissue-scale transport depends on geometric and transport parameters of the cells.

## Model of Hormone Transport in a File of Growing Cells with Spatially Varying Cell Lengths

### Discrete Model Set-up and Governing Equations

We now extend our model for hormone transport in a file of cells by allowing the cells to grow with time, and cell lengths and vacuolar fractions to vary along the file. Thus, in the discrete model, we prescribe each cell to have length ${\hat{l}}_{i}={\hat{l}}_{i}({\hat{t}})$ and vacuolar fraction $\phi _{i}=\phi _{i}({\hat{t}})$ that depend on time. For simplicity, we assume that, during growth, the width of the cells stays constant. We further assume the cell lengths do not become large enough that the timescale for cytoplasmic diffusion becomes comparable to the timescales for apoplastic diffusion and membrane transport, enabling us to maintain the assumption of spatially uniform concentrations within each compartment. Furthermore, if we assume growth is entirely due to enlargement of the vacuole, as is the case in the root elongation zone (Dolan and Davies 2004), then the volume of the cytoplasm in each cell, $(1-\phi _{i}) {\hat{l}}_{i}$, is constant in time and equal to the initial volume of the cytoplasm in each cell, respectively. For generality, we derive the full model without this assumption. Thus, the governing equations are

41a $$\begin{aligned} {\hat{w}} \dfrac{\textrm{d} }{\textrm{d} {\hat{t}}}\left( (1-\phi _{i}){\hat{l}}_{i} {\hat{c}}_{i}\right)&= {\hat{w}} ({\hat{J}}_{fci}-{\hat{J}}_{cfi}+{\hat{J}}_{cci}-{\hat{J}}_{cc(i-1)})-{\hat{l}}_{i} {\hat{J}}_{chi}-2\sqrt{\phi _{i}}({\hat{l}}_{i}+{\hat{w}}){\hat{J}}_{cvi}, \end{aligned}$$

41b $$\begin{aligned} {\hat{w}} \dfrac{\textrm{d} }{\textrm{d} {\hat{t}}}\left( \phi _{i} {\hat{l}}_{i} {\hat{v}}_{i}\right)&= 2\sqrt{\phi _{i}}({\hat{l}}_{i}+{\hat{w}}){\hat{J}}_{cvi}, \end{aligned}$$

41c $$\begin{aligned} {\hat{a}} {\hat{w}} \dfrac{\textrm{d} {\hat{f}}_{i}}{\textrm{d} {\hat{t}}}&= {\hat{w}} ({\hat{J}}_{cfi}-{\hat{J}}_{fc(i+1)})-{\hat{a}} {\hat{J}}_{fgi}, \end{aligned}$$

41d $$\begin{aligned} {\hat{a}}^2 \dfrac{\textrm{d} {\hat{g}}_{i}}{\textrm{d} {\hat{t}}}&= {\hat{a}}({\hat{J}}_{hgi}-{\hat{J}}_{gh(i+1)}+ {\hat{J}}_{fgi}), \end{aligned}$$

41e $$\begin{aligned} {\hat{a}} \dfrac{\textrm{d} }{\textrm{d} {\hat{t}}}\left( {\hat{l}}_{i} {\hat{h}}_{i}\right)&= {\hat{l}}_{i} {\hat{J}}_{chi} + {\hat{a}} ({\hat{J}}_{ghi}-{\hat{J}}_{hgi}), \end{aligned}$$

which is similar to (12), noting that ${\hat{l}}_{i}$ and $\phi _{i}$ are functions of time. The definitions of the fluxes are the same as in (11), except that ${\hat{l}}$ becomes ${\hat{l}}_{i}$. The system (41) is again subject to (13) and (14).

We non-dimensionalise the system using the following scales:

42 $$\begin{aligned}{} & {} {\hat{t}}=({\hat{L}}_{0}^2/{\hat{D}}_{\textrm{apo}})t, \quad ({\hat{c}}_{i},{\hat{v}}_{i},{\hat{f}}_{i},{\hat{g}}_{i},{\hat{h}}_{i})={\hat{C}}(c_{i}, v_{i}, f_{i}, g_{i}, h_{i}), \quad {\hat{l}}_{i}={\hat{l}}_{\textrm{av}} l_{i},\nonumber \\{} & {} \quad {\hat{J}}=\frac{{\hat{D}}_{\textrm{apo}} {\hat{C}}}{{\hat{l}}_{\textrm{av}}+{\hat{a}}}J, \end{aligned}$$

where ${\hat{L}}_{0}$ is the initial file length, and we define the average cell length at $t=0$, ${\hat{l}}_{\textrm{av}}$, by ${\hat{l}}_{\textrm{av}}={\hat{L}}_{0}/N-{\hat{a}}$. We note that the total file length is ${\hat{L}}({\hat{t}})=\sum _{i=1}^{N}({\hat{l}}_{i}({\hat{t}})+{\hat{a}})$.

The dimensionless equations are then

43a $$\begin{aligned} \frac{\epsilon ^2 \omega }{1+\lambda } \dfrac{\textrm{d} }{\textrm{d} t}\left( (1-\phi _{i}) l_{i} c_{i}\right)&= \omega (J_{fci}-J_{cfi}+J_{cci}-J_{cc(i-1)})-l_{i} J_{chi}-2\sqrt{\phi _{i}}(l_{i} + \omega )J_{cvi}, \end{aligned}$$

43b $$\begin{aligned} \frac{\epsilon ^2 \omega }{1+\lambda } \dfrac{\textrm{d} }{\textrm{d} t}\left( \phi _{i} l_{i} v_{i}\right)&= 2\sqrt{\phi _{i}}(l_{i}+\omega )J_{cvi}, \end{aligned}$$

43c $$\begin{aligned} \frac{\epsilon ^2 \lambda \omega }{1+\lambda } \dfrac{\textrm{d} f_{i}}{\textrm{d} t}&= \omega (J_{cfi}-J_{fc(i+1)})-\lambda J_{fgi}, \end{aligned}$$

43d $$\begin{aligned} \frac{\epsilon ^2 \lambda ^2}{1+\lambda } \dfrac{\textrm{d} g_{i}}{\textrm{d} t}&= \lambda (J_{hgi}-J_{gh(i+1)}+ J_{fgi}), \end{aligned}$$

43e $$\begin{aligned} \frac{\epsilon ^2 \lambda }{1+\lambda } \dfrac{\textrm{d} }{\textrm{d} t}\left( l_{i} h_{i}\right)&= l_{i} J_{chi} + \lambda (J_{ghi}-J_{hgi}), \end{aligned}$$

where $\epsilon , \lambda , \omega$ are given by (19), the fluxes *J* are defined by (17a-e,h), with ${\hat{l}}_{\textrm{av}}$ being used in the non-dimensionalisation instead of ${\hat{l}}$, and by

44a $$\begin{aligned} J_{ghi}&= \frac{2 (1+\lambda )}{l_{i}+\lambda } (g_{i-1}-h_{i}), \end{aligned}$$

44b $$\begin{aligned} J_{hgi}&= \frac{2 (1+\lambda )}{l_{i}+\lambda } (h_{i}-g_{i}). \end{aligned}$$

Again, (43) are subject to (20) and (21).

### Continuum Approximation

As in Sect. 2.2, we convert the discrete concentration variables into continuous variables that depend on both space and time, but this time with spatio-temporal variations in cell lengths. We also define continuous length and vacuolar-fraction functions

45 $$\begin{aligned} l(x,t)=l_{i}(t), \qquad \phi (x,t)=\phi _{i}(t), \end{aligned}$$

where we have non-dimensionalised distance along the file with the initial file length, ${\hat{x}}={\hat{L}}_{0} x$. Therefore, assuming that the cell lengths are slowly varying along the file and using Taylor’s theorem, we obtain, for example,

46 $$\begin{aligned} \begin{aligned} c_{i+1}(t)&=c\left( x+\frac{\epsilon (l_{i}+l_{i+1}+2\lambda )}{2(1+\lambda )},t\right) \\ {}&=c(x,t)+\epsilon \left( \frac{l+\lambda }{1+\lambda }\right) \dfrac{\partial c}{\partial x} \quad +\!\frac{\epsilon ^2}{2} \left( \frac{l\!+\!\lambda }{1\!+\!\lambda }\right) ^2 \dfrac{\partial ^2 c}{\partial x^2} \!+\! \frac{\epsilon ^2 (l\!+\!\lambda )}{2 (1\!+\!\lambda )^2} \dfrac{\partial l}{\partial x} \dfrac{\partial c}{\partial x} \!+\! O(\epsilon ^3) \end{aligned} \end{aligned}$$

for $\epsilon \ll 1$. The assumption of slow variation in the cell lengths means that we require $\partial l/ \partial x = O(1)$. We note that (46) is analogous to the expression in Band and King (2012), but, here, *l* may also depend on time. This is a significant feature of this model, since it means that the time derivatives in (43) are Lagrangian derivatives, which are related to the Eulerian time derivatives via

47 $$\begin{aligned} \dfrac{\textrm{d} }{\textrm{d} t}=\dfrac{\partial }{\partial t} + u\dfrac{\partial }{\partial x}, \end{aligned}$$

where *u*(*x*, *t*) is the velocity of the file at *x* relative to the position of the centre of the first cell at $x=0$. We can calculate *u* using conservation of mass in the file of cells (the continuity equation), which takes the form

48 $$\begin{aligned} \dfrac{\partial \rho }{\partial t} + \dfrac{\partial (\rho u)}{\partial x}=0, \end{aligned}$$

where $\rho (x,t)=1/(l+\lambda )$ is the cell density in the cell file. Thus, substituting for $\rho$ and rearranging,

49 $$\begin{aligned} \dfrac{\partial }{\partial x}\left( \frac{u}{l+\lambda }\right) =\frac{1}{(l+\lambda )^2}\dfrac{\partial l}{\partial t}. \end{aligned}$$

Therefore, given *l*(*x*, *t*), (49) can be integrated with respect to *x* to find *u*.

To derive the continuum approximation, we asymptotically expand all concentration variables in powers of $\epsilon$, as in (23). For simplicity, we write $l^{(0)}=l$ without further expanding the length function, *l*, since only the leading-order term contributes to the governing equations.

At leading order, we see that, in an analogous way to Sect. 2.5.1, the apoplastic and vacuolar concentrations are proportional to the cytoplasmic concentrations, as given in (25). This is because the structure of (43) is the same as (16), and it can be verified by substituting the relationships in (25) in the leading-order version of (43). Then, summing (43a)–(43e) to $O(\epsilon ^2)$, we obtain

50 $$\begin{aligned}{} & {} \frac{\epsilon ^2 }{1+\lambda } \dfrac{\textrm{d} }{\textrm{d} t}\left( \omega (1-\phi ) l c^{(0)} + \omega \phi l v^{(0)} + \lambda \omega f^{(0)} + \lambda ^2 g^{(0)} + \lambda l h^{(0)}\right) \nonumber \\{} & {} = \epsilon \left( \omega \left( -P_{\textrm{ac}} \dfrac{\partial f^{(0)}}{\partial x}+ P_{\textrm{ca}} \dfrac{\partial c^{(0)}}{\partial x}\right) \left( \frac{l+\lambda }{1+\lambda }\right) +2\lambda \left( -\dfrac{\partial g^{(0)}}{\partial x} + \dfrac{\partial h^{(0)}}{\partial x}\right) \right) \nonumber \\{} & {} + \frac{\epsilon ^2}{2} \left( \omega \left( P_{\textrm{ac}} \dfrac{\partial ^2 f^{(0)}}{\partial x^2}+ P_{\textrm{ca}}\dfrac{\partial ^2 c^{(0)}}{\partial x^2}+2P_{\textrm{plas}} \dfrac{\partial ^2 c^{(0)}}{\partial x^2}\right) \left( \frac{l+\lambda }{1+\lambda }\right) ^2\right. \nonumber \\{} & {} \left. +\,2\lambda \left( \dfrac{\partial ^2 g^{(0)}}{\partial x^2} + \dfrac{\partial ^2 h^{(0)}}{\partial x^2}\right) \left( \frac{l+\lambda }{1+\lambda }\right) \right) \nonumber \\{} & {} + \frac{\epsilon ^2}{2} \left( \omega \left( P_{\textrm{ac}} \dfrac{\partial f^{(0)}}{\partial x}+ P_{\textrm{ca}}\dfrac{\partial c^{(0)}}{\partial x}+2P_{\textrm{plas}} \dfrac{\partial c^{(0)}}{\partial x}\right) \frac{(l+\lambda )}{(1+\lambda )^2}\right. \nonumber \\{} & {} \left. +\,2\lambda \left( \dfrac{\partial g^{(0)}}{\partial x} + \dfrac{\partial h^{(0)}}{\partial x}\right) \frac{1}{1+\lambda } \right) \dfrac{\partial l}{\partial x} \nonumber \\{} & {} + \epsilon ^2 \left( \omega \left( -P_{\textrm{ac}} \dfrac{\partial f^{(1)}}{\partial x}+ P_{\textrm{ca}}\dfrac{\partial c^{(1)}}{\partial x}\right) \left( \frac{l+\lambda }{1+\lambda }\right) \right. \nonumber \\{} & {} \left. +\,2\lambda \left( -\dfrac{\partial g^{(1)}}{\partial x} + \dfrac{\partial h^{(1)}}{\partial x} + \left( g^{(1)}-h^{(1)}\right) \dfrac{\partial l}{\partial x} \frac{1}{l+\lambda } - \dfrac{\partial h^{(0)}}{\partial x} \dfrac{\partial l}{\partial x} \frac{1}{1+\lambda } \right) \right) ,\nonumber \\ \end{aligned}$$

where we note that we have additional terms, compared to (27), arising from the spatial dependence of *l*, and $\textrm{d}/\textrm{d}t$ is the Lagrangian derivative, as in (47). In particular, the third term on the right-hand side of (50) comes from the last term in the expansion (46) for the corresponding variables, and the last two terms in the fourth bracketed term on the right-hand side of (50) come from expanding $l_{i+1}$ in the definition (44a) of $J_{gh(i+1)}$ from (43d). As in Sect. 2.5.1, we observe that, because of (25), the $O(\epsilon )$ term in (50) is zero. To evaluate the last $O(\epsilon ^2)$ term on the right-hand side of (50), we use the analogous version of (28) (by summing (43a), (43b), and (43e) and considering the result at $O(\epsilon )$), which, in this case, takes the form

51 $$\begin{aligned} 0= & {} 2 \omega \left( P_{\textrm{ac}} f^{(1)}- P_{\textrm{ca}} c^{(1)}\right) +4\lambda \left( g^{(1)} - h^{(1)}\right) \left( \frac{1+\lambda }{l+\lambda }\right) \nonumber \\{} & {} - \left( \omega P_{\textrm{ac}} \dfrac{\partial f^{(0)}}{\partial x} \left( \frac{l+\lambda }{1+\lambda }\right) + 2\lambda \dfrac{\partial g^{(0)}}{\partial x}\right) . \end{aligned}$$

We note that (51) is obtained in an analogous way to (28), except that *l* is not equal to one, but is a function of *x* and *t*.

Differentiating (51) with respect to *x* and substituting into (50) gives

52 $$\begin{aligned}{} & {} \frac{\epsilon ^2 }{1+\lambda } \dfrac{\textrm{d} }{\textrm{d} t}\left( \omega (1-\phi ) l c^{(0)} + \omega \phi l v^{(0)} + \lambda \omega f^{(0)} + \lambda ^2 g^{(0)} + \lambda l h^{(0)}\right) \nonumber \\{} & {} = \frac{\epsilon ^2}{2} \left( \left( \omega P_{\textrm{ca}} \dfrac{\partial ^2 c^{(0)}}{\partial x^2} + 2 \omega P_{\textrm{plas}} \dfrac{\partial ^2 c^{(0)}}{\partial x^2}\right) \left( \frac{l+\lambda }{1+\lambda }\right) ^2+2\lambda \dfrac{\partial ^2 h^{(0)}}{\partial x^2}\left( \frac{l+\lambda }{1+\lambda }\right) \right) \nonumber \\{} & {} + \frac{\epsilon ^2}{2} \left( \omega P_{\textrm{ca}} \dfrac{\partial c^{(0)}}{\partial x} + 2 \omega P_{\textrm{plas}} \dfrac{\partial c^{(0)}}{\partial x} \right) \frac{(l+\lambda )}{(1+\lambda )^2} \dfrac{\partial l}{\partial x}. \nonumber \\ \end{aligned}$$

Thus, using (17a), (47), and writing $c^{(0)}=C$ for the macroscopic cytoplasmic concentration, we have

53 $$\begin{aligned} \left( \dfrac{\partial }{\partial t} + u\dfrac{\partial }{\partial x}\right) \left( (\omega (1-\phi ) l + \omega \phi l {\mathcal {P}}_{\textrm{v}} + \lambda (\omega + \lambda + l) {\mathcal {P}}_{\textrm{a}}) C \right){} & {} \nonumber \\ = (1+\lambda ) \left( \left( \frac{\omega P_{\textrm{ca}}}{2} + \omega P_{\textrm{plas}} \right) \left( \frac{l+\lambda }{1+\lambda }\right) ^2 + \lambda {\mathcal {P}}_{\textrm{a}} \left( \frac{l+\lambda }{1+\lambda }\right) \right) \dfrac{\partial ^2 C}{\partial x^2}{} & {} \nonumber \\ + (1+\lambda ) \left( \frac{\omega P_{\textrm{ca}}}{2} + \omega P_{\textrm{plas}} \right) \frac{(l+\lambda )}{(1+\lambda )^2} \dfrac{\partial l}{\partial x} \dfrac{\partial C}{\partial x}. \end{aligned}$$

This can be written in the standard form of an effective reaction–advection–diffusion equation

54 $$\begin{aligned} \dfrac{\partial C}{\partial t} + \dfrac{\partial }{\partial x}\left( U_{\textrm{eff}}(x,t) C\right) = \dfrac{\partial }{\partial x}\left( D_{\textrm{eff}}(x,t) \dfrac{\partial C}{\partial x}\right) - Q_{\textrm{eff}}(x,t) C, \end{aligned}$$

where

55a $$\begin{aligned} U_{\textrm{eff}}(x,t)&= u+\frac{K(l + \lambda )+M}{V} \dfrac{\partial l}{\partial x}-\frac{\left( K (l+\lambda )+M\right) (l+\lambda )}{V^2}\dfrac{\partial V}{\partial x}, \end{aligned}$$

55b $$\begin{aligned} D_{\textrm{eff}}(x,t)&= \frac{\left( K (l+\lambda )+M\right) (l+\lambda )}{V}, \end{aligned}$$

55c $$\begin{aligned} Q_{\textrm{eff}}(x,t)&= \frac{u}{V}\dfrac{\partial V}{\partial x} -\dfrac{\partial U_{\textrm{eff}}}{\partial x} +\frac{1}{V}\dfrac{\partial V}{\partial t}, \end{aligned}$$

and, for convenience, we have defined

56 $$\begin{aligned}{} & {} K=\frac{\omega P_{\textrm{ca}}/2 + \omega P_{\textrm{plas}}}{1 + \lambda }, \quad M=\lambda {\mathcal {P}}_{\textrm{a}}, \nonumber \\{} & {} V=\omega (1-\phi ) l + \omega \phi l {\mathcal {P}}_{\textrm{v}} + \lambda (\omega + \lambda + l) {\mathcal {P}}_{\textrm{a}}. \end{aligned}$$

This is subject to

57a $$\begin{aligned} C&=1 \quad \text { at } \quad x=0, \end{aligned}$$

57b $$\begin{aligned} \dfrac{\partial C}{\partial x}&=0 \quad \text { at } \quad x(t)=\sum _{i=1}^{N}(l_{i}+\lambda )/(N (1+\lambda )), \end{aligned}$$

57c $$\begin{aligned} C&=0 \quad \text { at } \quad t=0, \end{aligned}$$

noting that (57b) is the appropriate no-flux boundary condition given the moving boundary *x*(*t*).

To illustrate the generality of our model and identify its unique features, for comparison, in Appendix B, we present the simple cases of a file of cells that consist either of only a cytoplasmic compartment, or of cytoplasm with either a vacuolar or an apoplastic compartment. In summary, our analysis has revealed that spatio-temporal variations in cell length change the nature of the tissue-scale transport, as such variations create an effective advective velocity and an effective dilution (compare (54) with the continuum equation derived for the case of static identical cells (30)). To explore the model and the origin of these extra terms in detail, we first consider two base cases when *l* and $\phi$ are only time- or space-dependent (Sects. 3.3 and 3.4, respectively) before presenting numerical solutions in Sect. 3.6.

### The Case of Identical Time-Dependent Cell Lengths

As a particular case, we first consider a file of identical cells that grow with time, i.e., we take

58 $$\begin{aligned} {\hat{l}}_{i}({\hat{t}})={\hat{l}}({\hat{t}}), \qquad \phi _{i}({\hat{t}})=\phi ({\hat{t}}), \end{aligned}$$

where we non-dimensionalise the length with the initial cell length ${\hat{l}}_{0}={\hat{l}}_{0,i}={\hat{l}}_{\textrm{av}}$, since all lengths are the same. In this case, (49) can be solved to obtain

59 $$\begin{aligned} u(x,t)=\frac{x}{l+\lambda }\dfrac{\textrm{d} l}{\textrm{d} t}, \end{aligned}$$

i.e., velocity depends linearly on distance. In this case, (54) holds with

60 $$\begin{aligned}{} & {} U_{\textrm{eff}}(x,t) = u, \qquad D_{\textrm{eff}}(t) = \frac{\left( K (l+\lambda )+M\right) (l+\lambda )}{V}, \nonumber \\{} & {} \qquad Q_{\textrm{eff}}(t) = -\dfrac{\partial u}{\partial x} + \frac{1}{V}\dfrac{\textrm{d} V}{\textrm{d} t}, \end{aligned}$$

subject to (57), where (57b) is evaluated at $x=(l+\lambda )/(1+\lambda )$, and *K*, *M*, and *V* are defined by (56). We note that, here, cell growth induces an effective advective velocity and an effective sink term due to dilution.

We solve (54) with (57) and (60) numerically by first transforming to a fixed-interval domain. Thus, we set

61 $$\begin{aligned} x=\frac{l+\lambda }{1+\lambda } \xi , \end{aligned}$$

where $0 \le \xi \le 1$. Noting that

62a $$\begin{aligned} \dfrac{\partial }{\partial t}&=\dfrac{\partial }{\partial t} - \frac{\xi }{l+\lambda } \dfrac{\textrm{d} l}{\textrm{d} t} \dfrac{\partial }{\partial \xi }, \end{aligned}$$

62b $$\begin{aligned} \dfrac{\partial }{\partial x}&=\frac{1+\lambda }{l+\lambda } \dfrac{\partial }{\partial \xi }, \end{aligned}$$

(54) reads

63 $$\begin{aligned}&{} \dfrac{\partial C}{\partial t} = D_{\text {eff}}^{\text {cell}} \dfrac{\partial ^2 C}{\partial \xi ^2} - Q_{\text {eff}} C, \nonumber \\{}&{} D_{\text {eff}}^{\text {cell}}=\frac{(1+\lambda )^2 \left( K+M/(l+\lambda )\right) }{V}, \qquad Q_{\text {eff}}=\frac{1}{V}\dfrac{\text {d} V}{\text {d} t}, \end{aligned}$$

subject to

64a $$\begin{aligned} C&=1 \quad \text { at } \quad \xi =0, \end{aligned}$$

64b $$\begin{aligned} \dfrac{\partial C}{\partial \xi }&=0 \quad \text { at } \quad \xi =1, \end{aligned}$$

and (57c), where we define the inter-cellular diffusivity, $D_{\textrm{eff}}^{\textrm{cell}}$, in the fixed $\xi -$domain. We note that, as in the static case, we can identify points that are equally spread (1/*N* distance apart) in the $\xi$-domain, which is fixed, as the individual cells in the file.

For concreteness, we consider the case of linear cell growth, i.e.,

65 $$\begin{aligned} {\hat{l}}_{i}({\hat{t}})={\hat{l}}({\hat{t}})={\hat{k}} {\hat{t}}+{\hat{l}}_{0}, \end{aligned}$$

where ${\hat{k}}$ is the growth rate. Non-dimensionalising (65), we obtain

66 $$\begin{aligned} l_{i}(t)=l(t)=\kappa t+1, \end{aligned}$$

where $\kappa ={\hat{k}} {\hat{L}}_{0}^2/({\hat{D}}_{\textrm{apo}} {\hat{l}}_{0})$ measures the ratio of the growth rate and the diffusive rate on the cellular length scale. This could be thought of as a Péclet number, which can also be expressed in terms of the macroscopic length as $\kappa =(1+ \lambda ){\hat{k}} {\hat{L}}_{0}/(\epsilon {\hat{D}}_{\textrm{apo}})$.

When the cell growth rate is constant, i.e., $\textrm{d} l/\textrm{d} t=\kappa$ is constant, as in (66), we may seek a steady-state solution for the hormone concentration in the fixed $\xi$-domain when diffusion balances dilution due to growth over the macroscale level of the whole file. Noting that $M/(l+\lambda ) \rightarrow 0$ for large *t*, we solve the steady-state version of (63) to obtain

67 $$\begin{aligned} C_{\textrm{eq}}=\cosh {(\sqrt{\beta } \xi )} -\tanh {(\sqrt{\beta })} \sinh {(\sqrt{\beta } \xi )}, \end{aligned}$$

valid for large time, where, in the case of growth due to vacuolar expansion (see Sect. 3.6.1), $\beta =\kappa (\omega {\mathcal {P}}_{\textrm{v}} + \lambda {\mathcal {P}}_{\textrm{a}})/((1+\lambda ) (\omega P_{\textrm{ca}}/2 + \omega P_{\textrm{plas}} ))$ is a dimensionless parameter that measures the relative importance of growth and effective diffusive transport over the macroscale of the whole file. We note that we will obtain the same steady state if we only insist that $\textrm{d} l/\textrm{d} t \rightarrow \kappa - \textrm{constant}$ for large *t*, i.e., when the growth rate approaches a constant value as time increases. The existence of this non-trivial steady-state solution (in the $\xi$-domain) is a unique feature of this model, as, typically, growing-domain problems exhibit uniform steady-states (in the $\xi$-domain) due to constant diffusivity coefficients (see, for example, Simpson 2015).

### The Case of Spatially Varying Cell Lengths

Secondly, we consider a fixed file of cells with spatially varying lengths and vacuolar fractions

68 $$\begin{aligned} {\hat{l}}_{i}={\hat{l}}({\hat{x}}), \qquad \phi _{i}({\hat{x}})=\phi ({\hat{x}}), \end{aligned}$$

which we non-dimensionalise as in (42).

In practice, experimental datasets typically show the distribution of cell lengths (and vacuolar fractions) in terms of distance along the tissue (see, for example, Band et al. 2012). This can then be used to obtain a continuum representation of the cell-length distribution, ${\hat{l}}({\hat{x}})$, and the vacuolar-fraction distribution, $\phi ({\hat{x}})$.

Since the cells are non-growing, (49) gives

69 $$\begin{aligned} u=0. \end{aligned}$$

In this case, (54) holds, subject to (32) and (33), with

70 $$\begin{aligned} \begin{aligned} U_{\textrm{eff}}(x) = \frac{K(l + \lambda )+M}{V} \dfrac{\textrm{d} l}{\textrm{d} x}-\frac{\left( K (l+\lambda )+M\right) (l+\lambda )}{V^2}\dfrac{\textrm{d} V}{\textrm{d} x}, \\ D_{\textrm{eff}}(x) = \frac{\left( K (l+\lambda )+M\right) (l+\lambda )}{V}, \qquad Q_{\textrm{eff}}(x) = - \dfrac{\textrm{d} U_{\textrm{eff}}}{\textrm{d} x}. \end{aligned} \end{aligned}$$

We note that, here, unlike Sect. 3.3, it is the spatial variance in the cell lengths and vacuolar fractions that induces an effective advective velocity and an effective sink term.

Here, for concreteness, we prescribe linearly varying cell lengths on the macroscale of the file, i.e.,

71 $$\begin{aligned} {\hat{l}}({\hat{x}})=\hat{\alpha } {\hat{x}}+{\hat{l}}_{1}, \end{aligned}$$

where $\hat{\alpha }$ is a proportionality constant, and ${\hat{l}}_{1}$ is the length of the first cell. This distribution is appropriate for cells in the *Arabidopsis* root elongation zone (see, for example, Band et al. 2012). Non-dimensionalising (71), we obtain

72 $$\begin{aligned} l(x)=\alpha x+\nu , \end{aligned}$$

where $\alpha =({\hat{l}}_{N+1}-{\hat{l}}_{1})/{\hat{l}}_{\textrm{av}}$ and $\nu ={\hat{l}}_{1}/{\hat{l}}_{\textrm{av}}$. Similarly, we prescribe the macroscale distribution of the vacuolar fraction,

73 $$\begin{aligned} \phi (x)=(\phi _{N+1}-\phi _{1}) x+\phi _{1}. \end{aligned}$$

In order to obtain the corresponding values for ${\hat{l}}_{i}$ (and $l_{i}$), we need to discretise (71) at the centre of each cell. For the purposes of the numerical solutions, we specify the lengths and vacuolar fractions of the first and the last cell, and we find that this, together with (72) is enough to uniquely determine the lengths and vacuolar fractions of each cell, the average cell length, ${\hat{l}}_{\textrm{av}}$, and the file length, ${\hat{L}}$. In particular, when calculating $l_{i}$ and $\phi _{i}$, we evaluate *l*(*x*) and $\phi (x)$, respectively, at the centre $x_{i}$ of cell *i*, which can be calculated iteratively from

74 $$\begin{aligned} x_{i}=\frac{1}{N (1+\lambda )} \left( \frac{l_{1}}{2} + \sum _{j=2}^{i-1}l_{j} + \frac{l_{i}}{2} + (i-1) \lambda \right) , \end{aligned}$$

where the last term comes from the contribution of the apoplast thickness.

### The Origin of the Induced Effective Velocity due to Spatially Varying Cell Lengths

In the case of identical growing cells (Sect. 3.3), an effective velocity is naturally induced due to cell growth. However, the origin of the induced velocity in the case of non-growing cells ($u=0$) with spatially varying lengths and vacuolar fractions is less intuitive and requires the further analysis presented below.

As shown in Appendix B, for a file of cells comprised of only cytoplasmic compartments, having spatially varying cell lengths does not lead to an induced effective advective velocity. However, introducing either vacuolar compartments or apoplastic compartments alone is sufficient to induce an advective velocity. Furthermore, in these two simpler cases, the induced velocity is proportional to both the spatial gradient of the vacuolar fraction or the cell lengths, respectively, and ${\mathcal {P}}_{\textrm{v}}-1$ or ${\mathcal {P}}_{\textrm{a}}-1$, respectively. Thus, not only is spatial variance in cell lengths necessary for an induced velocity, but also the presence of unequal flow rates out of and into the cytoplasm.

To explain these results, we consider the ratio between the cytoplasmic hormone concentration and the area-weighted average concentration, $C/{\mathcal {C}}$, where the area-weighted average concentration is defined by

75 $$\begin{aligned} {\mathcal {C}}(x,t)=\frac{(1-\phi ) \omega l c^{(0)} + \phi \omega l v^{(0)} + \lambda \omega f^{(0)} + \lambda ^2 g^{(0)} + \lambda l h^{(0)}}{{\mathcal {V}}}, \end{aligned}$$

where

76 $$\begin{aligned} {\mathcal {V}}=(l+\lambda )(\omega +\lambda ) \end{aligned}$$

is the dimensionless area of each cell. Using (25), we can relate the average concentration to the cytoplasmic one via

77 $$\begin{aligned} {\mathcal {C}}=\frac{(1-\phi ) \omega l + \phi \omega l {\mathcal {P}}_{\textrm{v}} + \lambda (\omega + \lambda + l) {\mathcal {P}}_{\textrm{a}}}{{\mathcal {V}}} C. \end{aligned}$$

Considering (56) (with $l=l(x)$ for non-growing cells), we see that

78 $$\begin{aligned} \frac{C}{{\mathcal {C}}}=\frac{{\mathcal {V}}}{V}. \end{aligned}$$

If $C/{\mathcal {C}}$ increases with distance along the cell file, i.e., its derivative with respect to *x* is positive, this means that the relative proportion of cytoplasmic hormone concentration in the cells increases, and therefore we interpret this as an effective velocity being induced in the same direction and vice versa. In the particular case of cells having only cytoplasmic and vacuolar compartments (without apoplast, i.e., $\lambda =0$), after rearrangement, we calculate

79 $$\begin{aligned} \frac{C}{{\mathcal {C}}}=\frac{1}{1+\phi ({\mathcal {P}}_{\textrm{v}}-1)}, \end{aligned}$$

and so

80 $$\begin{aligned} \dfrac{\textrm{d} }{\textrm{d} x}\left( \frac{C}{{\mathcal {C}}}\right) =-\dfrac{\textrm{d} \phi }{\textrm{d} x}\frac{{\mathcal {P}}_{\textrm{v}}-1}{(1+\phi ({\mathcal {P}}_{\textrm{v}}-1))^2}. \end{aligned}$$

Thus, in the case of GA for a file of cells with increasing vacuole sizes, a negative velocity is induced, since ${\mathcal {P}}_{\textrm{v}}>1$ (from (26)), which is in agreement with the analysis in Appendix B. Similarly, in the case of cells having only cytoplasmic and apoplastic compartments (without a vacuole, i.e., $\phi =0$), we find

81 $$\begin{aligned} \frac{C}{{\mathcal {C}}}=\frac{(l+\lambda )(\omega +\lambda )}{\omega l+\lambda (\omega +\lambda +l) {\mathcal {P}}_{\textrm{a}}}, \end{aligned}$$

and so

82 $$\begin{aligned} \dfrac{\textrm{d} }{\textrm{d} x}\left( \frac{C}{{\mathcal {C}}}\right) =-\dfrac{\textrm{d} l}{\textrm{d} x} \frac{\omega \lambda (1-{\mathcal {P}}_{\textrm{a}}) (\omega +\lambda )}{(\omega l+\lambda (\omega +\lambda +l) {\mathcal {P}}_{\textrm{a}})^2}. \end{aligned}$$

Again, in the case of GA for a file of cells with increasing lengths, a negative velocity is induced, since ${\mathcal {P}}_{\textrm{a}}<1$ (from (26)), which is also in agreement with the results in Appendix B. It is worth noting that, in the particular case, when there are only “longitudinal” apoplastic compartments (i.e., there are no apoplastic compartments between cell cytoplasms), then we find that $C/{\mathcal {C}}=(\omega +\lambda )/(\omega +\lambda {\mathcal {P}}_{\textrm{a}})$, which does not change with *x*, and, consequently, there is no induced velocity. However, if we keep the “transverse” apoplastic compartments only, then

83 $$\begin{aligned} \frac{C}{{\mathcal {C}}}=\frac{l+\lambda }{l+\lambda {\mathcal {P}}_{\textrm{a}}}, \end{aligned}$$

and so

84 $$\begin{aligned} \dfrac{\textrm{d} }{\textrm{d} x}\left( \frac{C}{{\mathcal {C}}}\right) =-\dfrac{\textrm{d} l}{\textrm{d} x} \frac{\lambda (1-{\mathcal {P}}_{\textrm{a}})}{(l+\lambda {\mathcal {P}}_{\textrm{a}})^2}. \end{aligned}$$

Thus, the “transverse” apoplastic compartments alone are enough to induce negative effective velocity.

This analysis suggests that considering the sign of the derivative of $C/{\mathcal {C}}$ is a faithful criterion in determining whether there is an induced effective velocity and what its direction is. Indeed, if we consider the expression for the effective velocity (70), we see that it can be rewritten as

85 $$\begin{aligned} U_{\textrm{eff}}(x) = \frac{K(l + \lambda )+M}{\omega +\lambda } \dfrac{\textrm{d} }{\textrm{d} x}\left( \frac{{\mathcal {V}}}{V}\right) =\frac{K(l + \lambda )+M}{\omega +\lambda } \dfrac{\textrm{d} }{\textrm{d} x}\left( \frac{C}{{\mathcal {C}}}\right) , \end{aligned}$$

using (78). Therefore, the sign of the spatial derivative of $C/{\mathcal {C}}$ coincides with the sign of the induced velocity. Thus, we may now use this to study the more complicated general case when there are both a vacuolar and apoplastic compartments in the cells. In this case, (78) holds, and thus

86 $$\begin{aligned}{} & {} \dfrac{\textrm{d} }{\textrm{d} x}\left( \frac{C}{{\mathcal {C}}}\right) =-\frac{\omega (\omega +\lambda )}{((1-\phi ) \omega l + \phi \omega l {\mathcal {P}}_{\textrm{v}} + \lambda (\omega + \lambda + l) {\mathcal {P}}_{\textrm{a}})^2}\nonumber \\{} & {} \left( \left( l^2 \dfrac{\textrm{d} \phi }{\textrm{d} x}+\lambda l \dfrac{\textrm{d} \phi }{\textrm{d} x} + \lambda \phi \dfrac{\textrm{d} l}{\textrm{d} x}\right) ({\mathcal {P}}_{\textrm{v}}-1) + \lambda \dfrac{\textrm{d} l}{\textrm{d} x} (1-{\mathcal {P}}_{\textrm{a}})\right) . \end{aligned}$$

In the case of GA for a file of cells of increasing cell lengths and vacuolar fractions, and with ${\mathcal {P}}_{\textrm{v}}>1, {\mathcal {P}}_{\textrm{a}}<1$, the right-hand side of (86) is negative, and thus a negative effective velocity is induced, decreasing the rate of hormone transport. We note that (80) and (82) can be obtained from (86) by setting $\phi =0$ and $\lambda =0$, respectively. We also observe that, if the cell lengths and the vacuolar fractions decrease along the cell file, then a positive effective velocity is induced.

From (86), it is evident that the presence of a vacuole alters the transport dynamics, regardless of the associated tonoplast transporter. For example, even if the cell lengths increase with *x* and ${\mathcal {P}}_{\textrm{v}}>1$, a positive velocity can still be induced if the vacuolar fractions decrease with *x* at a sufficiently large rate. For the purposes of concreteness, we consider the case when the cytoplasmic area, $\sigma = (1-\phi ) l$, is constant among all cells, i.e., the spatial variance is entirely due to changes in the size of the vacuole, as would be the case in the root elongation zone. This means that

87 $$\begin{aligned} \dfrac{\textrm{d} }{\textrm{d} x}\left( (1-\phi ) l\right) =(1-\phi ) \dfrac{\textrm{d} l}{\textrm{d} x} - l(x) \dfrac{\textrm{d} \phi }{\textrm{d} x}=0. \end{aligned}$$

Substituting (87) into (86), we obtain

88 $$\begin{aligned} \dfrac{\textrm{d} }{\textrm{d} x}\left( \frac{C}{{\mathcal {C}}}\right) =\frac{\omega (\omega +\lambda ) ((\sigma + \lambda ) ({\mathcal {P}}_{\textrm{v}}-1) + \lambda (1-{\mathcal {P}}_{\textrm{a}}))}{((1-\phi ) \omega l + \phi \omega l {\mathcal {P}}_{\textrm{v}} + \lambda (\omega + \lambda + l) {\mathcal {P}}_{\textrm{a}})^2} \dfrac{\textrm{d} l}{\textrm{d} x}. \end{aligned}$$

Normally, for GA, ${\mathcal {P}}_{\textrm{a}}<1$ even when the cytoplasmic membrane transporter is absent, as in this case, ${\mathcal {P}}_{\textrm{a}}=B_{1}/A_{1} \ll 1$. However, if the tonoplast transporter is absent, ${\mathcal {P}}_{\textrm{v}}=B_{1}/C_{1} \ll 1$, and so, provided the cell lengths increase with *x*, a positive effective velocity is induced when

89 $$\begin{aligned} {\mathcal {P}}_{\textrm{v}} < \frac{\sigma + \lambda {\mathcal {P}}_{\textrm{a}}}{\sigma + \lambda }, \end{aligned}$$

which is thus satisfied when the tonoplast transporter is absent, since the right-hand side of (89) is close to one for small $\lambda$ and ${\mathcal {P}}_{\textrm{a}}$. This is expected, since, when there is no tonoplast transporter, GA is not taken up by the vacuole and is free to diffuse along the file. Less intuitive, however, is the fact that a negative velocity can be induced for a small interval of values for $(\sigma +\lambda {\mathcal {P}}_{\textrm{a}})/(\sigma + \lambda )<{\mathcal {P}}_{\textrm{v}}<1$ when GA is still readily transported out of the vacuole. This is a result of the cytoplasmic membrane transporters competitively interacting with the tonoplast transporters when ${\mathcal {P}}_{\textrm{a}}<1$.

### Numerical Results

We now present numerical simulations of the two base cases considered in Sects. 3.3 and 3.4 comparing the discrete and continuum formulations. In both cases, we use *Mathematica* to solve the discrete model and use the method of lines with 100 grid points in space to solve the continuum model. We then use the continuum model to investigate the behaviour of the system further and how it depends on the physical parameters.

#### Results for a File of Identical Growing Cells

We now solve the discrete model (43) with (17a–17e) and (44), subject to (20) and (21) with (66), where ${\hat{l}}_{0}={\hat{l}}_{0,i}={\hat{l}}_{\textrm{av}}=20 \, \upmu \textrm{m}$, $\phi _{0}=\phi _{0,i}=0.1$, and $P_{\textrm{plas}}=0$. We further assume that cell growth is due to vacuolar expansion, as is the case for cells located in the root elongation zone. This means that the cytoplasmic area, $(1-\phi )l$, is constant in time, and thus $\phi =1-(1-\phi _{0})l_{0}/l$, where $l_{0}=l_{0,i}$ and $\phi _{0}=\phi _{0,i}$ are the initial cell lengths and vacuolar fractions, respectively. In this case, $\textrm{d} V/\textrm{d} t=(\omega {\mathcal {P}}_{\textrm{v}}+\lambda {\mathcal {P}}_{\textrm{a}})\textrm{d} l/ \textrm{d} t.$ We choose $\kappa =0.01$ to illustrate the qualitative behaviour of the solutions when the effect of diffusion on GA transport over the file of cells is moderately comparable to the effect of cell growth. For the continuum model, we solve (63), subject to (57c) and (64).

**Fig. 4:**
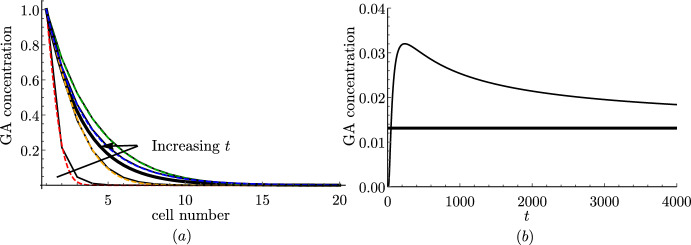
Continuum model predictions for growing identical cells agree with discrete model. **a** Plots of the spatial profile of the cytoplasmic GA concentration *c* from the discrete model (solid black) and the macroscopic cytoplasmic GA concentration *C* from the continuum model (dashed) at each cell for $t=1$ (red), 10 (orange), 100 (green), 1000 (blue) and constant growth rate $\kappa =0.01$, and the steady-state solution $C_{\textrm{eq}}$ from (67) (thick black), **b** temporal profile of the macroscopic cytoplasmic GA concentration *C* at the middle of the file (tenth cell) and the corresponding concentration value of the steady-state concentration $C_{\textrm{eq}}$ from (67) (thick black). Here, ${\hat{l}}_{0}=20 \, \upmu \textrm{m}$, $\phi _{0}=0.1$, and $P_{\textrm{plas}}=0$ (Color figure online)

We see an excellent agreement between the discrete and the continuum solutions, which approach the steady solution, (67), for large time (Fig. 4a). The profiles of the concentration are monotonically decreasing along the file. Within each cell, the concentration initially increases with time, but, after a finite time, it starts to decrease (Fig. 4a, b). This is because, in this regime, dilution due to growth gradually becomes a more dominant effect than diffusion, driving the GA concentration down until a balance between the two processes is reached, indicated by the steady-state profile.

**Fig. 5:**
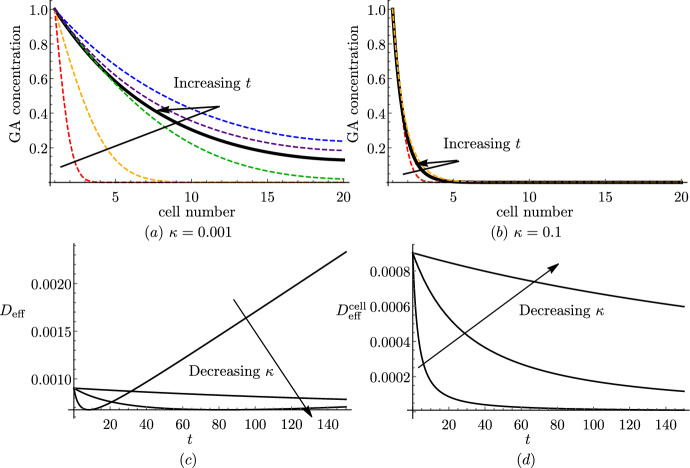
Effect of the constant growth rate, $\kappa$, on the predicted GA concentration for growing identical cells. Plots of the spatial profile of the macroscopic cytoplasmic GA concentration *C* from the continuum model at each cell for **a** $t=1$ (red), 10 (orange), 100 (green), 1000 (blue), 10,000 (purple) and $\kappa =0.001$, and **b** $t=1$ (red), 100 (orange) and $\kappa =0.1$, together with the steady-state solution $C_{\textrm{eq}}$ from (67) (thick black). Plots of the temporal profile of the **c** effective diffusivity as from (60) and **d** the effective inter-cellular diffusivity as from (63) for $\kappa =0.001, 0.01, 0.1$. Here, ${\hat{l}}_{0}=20 \, \upmu \textrm{m}$, $\phi _{0}=0.1$, and $P_{\textrm{plas}}=0$ (Color figure online)

We see that, for smaller values of the constant dimensionless growth rate $\kappa$, GA spreads to the end of the file and raises the concentration in all cells in the long-term, as indicated by the steady-state profile (Fig. 5a, b). However, for larger values of $\kappa$, GA concentration rises only in the first few cells, remaining zero everywhere else. This is because, with a smaller $\kappa$, transport by diffusion is more dominant over the effect of dilution due to growth, and, therefore, GA is able to reach farther along the file. The relative effects of dilution and diffusion on the macroscale is represented by the parameter $\beta$: we note that $\kappa =0.001$ corresponds to $\beta =7.5=O(1)$, and, indeed, we see a balance between these two effects over the length of the whole file.

Considering how the effective diffusivity, (60), varies with time for different values of $\kappa$ (Fig. 5c), we see that, after an initial decrease, due to competition between the effects of the cytoplasmic and apoplastic contributions, the effective diffusivity becomes monotonically increasing with time. This reflects our previous observation for non-growing cells in Sects. 2.5.2 and 2.5.3 that the effective diffusivity is larger for long cells. This is also the reason why, at later times, for smaller values of $\kappa$ (i.e. smaller values of the growth rate, ${\hat{k}}$), the effective diffusivity is smaller. In Fig. 5d, we show how the effective inter-cellular diffusivity from (63) in the fixed $\xi$-domain varies with time for the same values of $\kappa$. In this case, this diffusivity measures how fast GA is transported from one cell to another, rather than across a given distance. Thus, this effective diffusivity decreases with time, since the cells grow, and the effective diffusivity is larger for smaller values of $\kappa$, as this corresponds to a slower growth rate.

#### Results for a File of Spatially Varying Static Cells

We now solve the discrete model (43) with (17a–17e), and (44), subject to (20) and (21) with (72–74), where ${\hat{l}}_{1}=20 \, \upmu \textrm{m}$, ${\hat{l}}_{N+1}=200 \, \upmu \textrm{m}$, $\phi _{1}=0.1$, $\phi _{N+1}=0.9$, and $P_{\textrm{plas}}=0$ (choosing parameter values appropriate for a cell file along the *Arabidopsis* root elongation zone (Band et al. 2012)). We find that ${\hat{l}}_{\textrm{av}}\approx 78.5 \, \upmu \textrm{m}$ and ${\hat{L}} \approx 1580 \, \upmu \textrm{m}$, and thus $\alpha =2.29$ and $\nu = 0.25$. For the continuum model, we solve (54) with (70), subject to (32) and (33).

**Fig. 6:**
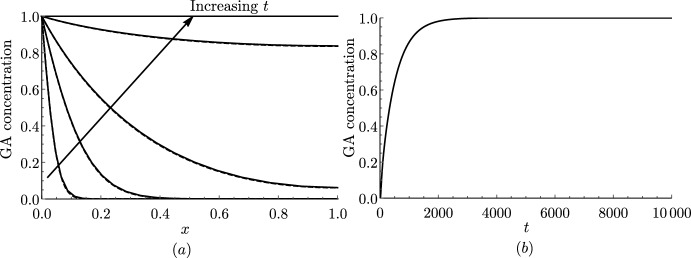
Continuum model predictions for non-growing cells with spatially varying lengths agree with discrete model. **a** Plots of the spatial profile of the cytoplasmic GA concentration *c* from the discrete model (solid black) and the macroscopic cytoplasmic GA concentration *C* from the continuum model (dashed black) for $t=1, 10, 100, 1000, 10,000$, and **b** temporal profile of the macroscopic cytoplasmic GA concentration *C* at the middle of the file ($x=0.5$), where $\alpha = 2.29$, and $\nu = 0.25$

Comparing of the GA distributions from the discrete and the continuum models for different times, we see an excellent agreement for all times (Fig. 6a). We note that the agreement for early time is better than the case of static identical cells (compare Fig. 6a and 2a). This is because, in the case of spatially varying cell lengths, there are more cells in the part of the file where there is a steep gradient in the GA concentration profile at early times. As expected, at large times, the GA concentrations approach a uniform steady state (Fig. 6a, b).

**Fig. 7:**
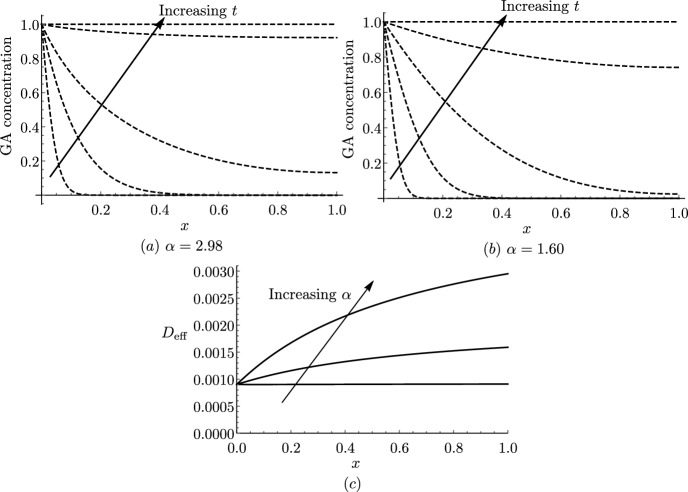
Effect of cell-length gradient on the GA concentrations and effective diffusivity. Plots of the spatial profile of the macroscale cytoplasmic GA concentration *C* from the continuum model for $t=1, 10, 100, 1000, 10000$ for **a** $\alpha =2.98$ (final cell length is $400 \, \upmu \textrm{m}$) and **b** $\alpha =1.60$ (final cell length is $100 \, \upmu \textrm{m}$). **c** Plots of the spatial profile of the effective diffusivity as from (70) for $\alpha =1.60, 2.29, 2.98$ (and final cell lengths $100, 200, 400 \, \upmu \textrm{m}$, respectively) and $\nu =0.40, 0.25, 0.16$

In Fig. 7a and b, we show spatial plots of the macroscopic GA concentration for cell files with final cell length of $400 \, \upmu \textrm{m}$ and $100 \, \upmu \textrm{m}$, respectively. In the first case, we find that ${\hat{l}}_{\textrm{av}} \approx 127.6 \, \upmu \textrm{m}$, ${\hat{L}} \approx 2562 \, \upmu \textrm{m}$, $\alpha =2.98$, $\nu = 0.16$, and, in the second case, ${\hat{l}}_{\textrm{av}} \approx 49.85 \, \upmu \textrm{m}$, ${\hat{L}} \approx 1007 \, \upmu \textrm{m}$, $\alpha =1.60$, $\nu = 0.40$. We see that, increasing the final cell length (via an increase in $\alpha$), leads to a faster approach to the steady state. This is consistent with the results for static identical cells, where, in the case of long cells, the effective diffusivity is larger than in the case of short cells, and thus the GA concentration approaches the steady state faster. We note that the value of $\alpha$ affects how the effective diffusivity varies with distance along the cell file (Fig. 7c). With smaller $\alpha$, the effective diffusivity decreases and becomes more uniform across the file. In fact, for the case of a final cell length of $100 \, \upmu \textrm{m}$, i.e., five times longer than the initial cell length, the effective diffusivity is almost constant.

Finally, we explore how the velocity induced by the spatial variance in cell lengths along the file affects the hormone transport dynamics. We consider three different cases, namely, a file of cells with increasing lengths and vacuolar fractions, i.e., ${\hat{l}}_{1}=20 \, \upmu \textrm{m}$, ${\hat{l}}_{N+1}=200 \, \upmu \textrm{m}$, $\phi _{1}=0.1$, $\phi _{N+1}=0.9$, and $P_{\textrm{plas}}=0$, a file of cells that is the mirror image of this one with respect to its middle, i.e., ${\hat{l}}_{1}=200 \, \upmu \textrm{m}$, ${\hat{l}}_{N+1}=20 \, \upmu \textrm{m}$, $\phi _{1}=0.9$, $\phi _{N+1}=0.1$, and a file, which consists of identical cells of length equal to the average length in the previous two cases, i.e., ${\hat{l}}_{\textrm{av}}\approx 78.5 \, \upmu \textrm{m}$. This will allow for a fair comparison, because we keep the same number of cells and the total length of the file. To be consistent, we also need to choose an average vacuolar fraction, $\phi _{\textrm{av}}$, for all cells in the third case, and we do this by insisting on preserving the total vacuolar area in the file, i.e.,

90 $$\begin{aligned} \frac{\phi _{1} l_{1}}{2} + \sum _{j=2}^{N}\phi _{j} l_{j} + \frac{\phi _{N+1} l_{N+1}}{2} = N \phi _{\textrm{av}} l_{\textrm{av}}. \end{aligned}$$

We find that $\phi _{\textrm{av}} \approx 0.5$.

**Fig. 8:**
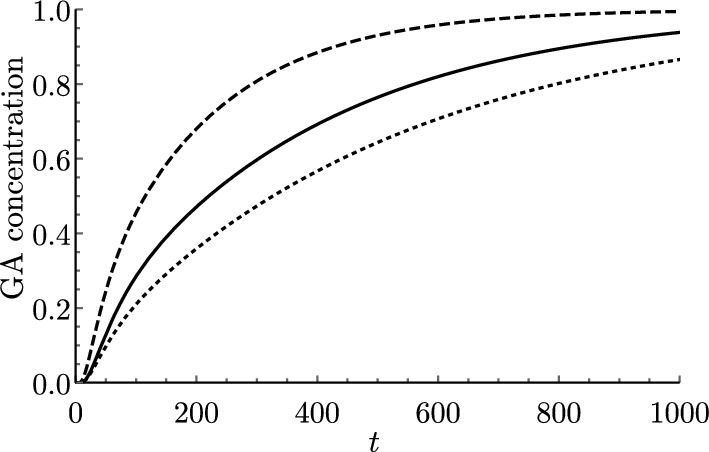
GA is transported faster in a file of cells with decreasing lengths compared to a file of cells with increasing lengths. Plots of the temporal profile of the GA concentration at the middle of the file, $x=0.5$, for a file of identical cells (solid black), decreasing cell lengths (dashed), and increasing cell lengths (dotted)

In Fig. 8, we show the temporal profiles of the GA concentration at the middle of the file for these three cases. We see that, in the case of decreasing cell lengths, transport is fastest, and the steady state $C=1$ is reached at $t=1000$, whereas the slowest transport occurs in the case of a file of cells of increasing lengths. The transport rate in the case of identical cell lengths is between the other two cases. This is consistent with our analysis in Sect. 3.5, since we found that, when the cell lengths are decreasing, this induces a positive advective velocity, which promotes hormone transport along the file. For example, looking at the times when $C=0.5$ is achieved, we see that it takes twice and 3.5 times as long in the case of identical cell lengths and the case of increasing cell lengths, respectively, compared to the case of decreasing cell lengths.

## Modelling the Effect of Cell Division

Within biological tissues, growing cells will typically divide at regular intervals to maintain approximately constant cell sizes. Such a combination of growth and division is typically essential for cellular functioning, as biochemical processes may break down if cells became excessively large. Motivated by this, we now incorporate cell division into our model for identical growing cells, in order to understand what effect this has on the tissue-scale hormone transport. In this section, we aim to capture more realistic cell growth dynamics and assume an exponential cell growth, such that the relative elongation rate, ${\hat{r}}$, of the cells is constant (Beemster and Baskin 1998; Green 1976). In particular, using the non-dimensionalisation from Sect. 3.3, the dimensionless cell lengths, *l*(*t*), satisfy

91 $$\begin{aligned} \frac{1}{l}\dfrac{\textrm{d} l}{\textrm{d} t}=\mu , \end{aligned}$$

where $\mu ={\hat{r}} {\hat{L}}^2_{0}/{\hat{D}}_{\textrm{apo}}$ is the dimensionless relative elongation rate. We assume that the cells divide synchronously at a constant cell division rate such that, as soon as a cell doubles in size, it divides in two equal cells. Thus, we require the inter-division time, *T* to be related to the relative elongation rate via

92 $$\begin{aligned} \mu =\frac{\log {(2)}}{T}. \end{aligned}$$

as detailed in Green (1976). Furthermore, since vacuoles remain small in the meristem (Rizza et al. 2021), we assume that cell growth occurs due to both cytoplasmic and vacuolar expansion such that the vacuoles remains at $10\%$ of the cells area during growth and division (thus, $\phi =0.1$ is constant).

Assuming that that every cell follows the exponential growth, given by (91), and at every cell division (occurring at every *T* time units), the current length is partitioned between two equal lengths of the two new cells and one thickness of a new apoplastic compartment, located between the two cells, we calculate the cell-length function $l_{\textrm{d}}$ to be

93 $$\begin{aligned} l_{\textrm{d}}=\left( 1-\lambda \left( 1-2^{-\lfloor t/T \rfloor }\right) \right) \textrm{e}^{\mu (t-T \lfloor t/T \rfloor )}, \end{aligned}$$

where $\lfloor . \rfloor$ is the floor function. We note that $l_{\textrm{d}}$ is discontinuous at the points of cell division, namely, $t=nT$, where $n \in {\mathbb {N}}$. Thus, the discrete model (43), subject to (20) and (21), is valid everywhere except at the points of cell division.

The governing continuum equation is (54) with (60), as in Sect. 3.3, valid again everywhere except at the points of cell division, where, now

94 $$\begin{aligned} U_{\textrm{eff}}(x,t)= & {} u_{\textrm{d}}=\frac{x}{l_{\textrm{d}}+\lambda }\dfrac{\textrm{d} l_{\textrm{d}}}{\textrm{d} t}, \qquad D_{\textrm{eff}}(t) = \frac{\left( K (l_{\textrm{d}}+\lambda )+M\right) (l_{\textrm{d}}+\lambda )}{V_{\textrm{d}}}, \nonumber \\ Q_{\textrm{eff}}(t)= & {} -\dfrac{\partial u_{\textrm{d}}}{\partial x} + \frac{1}{V_{\textrm{d}}}\dfrac{\textrm{d} V_{\textrm{d}}}{\textrm{d} t}. \end{aligned}$$

Here, $V_{\textrm{d}}$ is the equivalent expression for *V*, accounting for cell division, and is thus given by

95 $$\begin{aligned} V_{\textrm{d}}=\omega (1-\phi ) l_{\textrm{d}} + \omega \phi l_{\textrm{d}} {\mathcal {P}}_{\textrm{v}} + \lambda (\omega + \lambda + l_{\textrm{d}}) {\mathcal {P}}_{\textrm{a}}. \end{aligned}$$

This time (54) with (94) is subject to (57), where (57b) is evaluated at $x=(l+\lambda )/(1+\lambda )$, where *l* is given through (91), as division does not affect the growth of the whole file of cells. When numerically solving (54) with (94), we employ the same method as in Sect. 3.3 and transform to a fixed-interval domain using (61). We treat the case of cells with a single compartment in Appendix B.

**Fig. 9:**
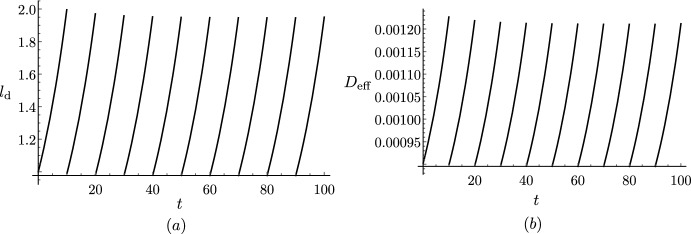
Cell division and growth together can induce periodic behaviour in cell lengths and diffusivity. Plots of the temporal profile of the **a** cell lengths, $l_{\textrm{d}}$, and **b** effective diffusivity, $D_{\textrm{eff}}$, with $T=10$, $\phi =0.1$, and $P_{\textrm{plas}}=0$

In Fig. 9a, we show a temporal plot of the cell lengths $l_{\textrm{d}}$ for the case when $T=10$. We see that the lengths exhibit periodic behaviour due to the counteracting effects of growth and cell division. Similarly, in Fig. 9b, we show a temporal plot of the corresponding effective diffusivity, $D_{\textrm{eff}}$, given in (94). We again see that it eventually settles into a periodic behaviour due to the periodicity in $l_{\textrm{d}}$.

**Fig. 10:**
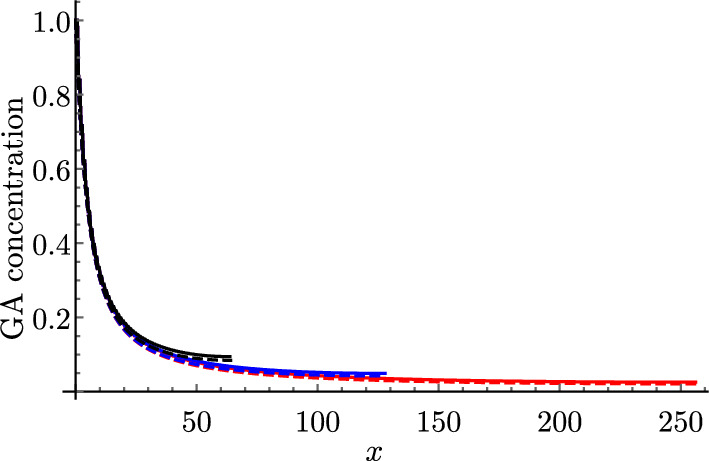
Continuum model predictions for growing and dividing cells agree with discrete model. Plots of the spatial profile of the cytoplasmic GA concentration *c* from the discrete model (solid lines) and the macroscopic cytoplasmic GA concentration *C* from the continuum model (dashed lines) after five (black), six (blue), and seven (red) cell divisions. Here, the inter-division time is 10 h, $\phi =0.1$, and distance *x* is non-dimensionalised with the initial length of the file (Color figure online)

In Fig. 10, we show a comparison between the spatial profiles of the GA concentration in the discrete and continuum models after six, seven, and eight synchronous cell divisions, respectively, starting with a file of two cells of length $5 \, \upmu \textrm{m}$ each. We see an excellent agreement confirming that the continuum model with the modified cell length accurately represents the transport dynamics in the discrete system.

**Fig. 11:**
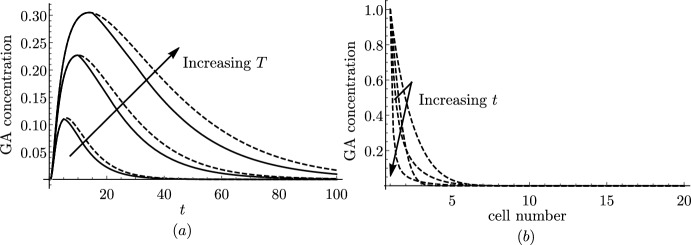
Decreasing the relative elongation rate through an increase in the inter-division time promotes the effect of cell division, which acts to slow down GA transport. **a** Plots of the temporal profile of the GA concentration at a fixed point in the file, $x=0.1$, for different inter-division times $T=5, 10, 15$ (solid black) and the corresponding plots without cell division (dashed black). **b** Plots of the spatial profile of the GA concentration for $t=1, 10, 30, 50$ with $T=10$. The cell numbers on the axis reflect the initial number of cells. In all plots, $\phi =0.1$ and $P_{\textrm{plas}}=0$

In Fig. 11a, we show a temporal profile of the GA concentration at a fixed point ($x=0.1$) along the file for different inter-division times *T*, compared to the corresponding plots without cell division (but with the same corresponding relative elongation rate). We see that all profiles are similar to Fig. 4b, except that, here, they decrease down to zero due to the exponential form of the growth, which, unlike linear growth, allows only for a trivial steady state. Furthermore, we see that, as the inter-division time increases, the concentration increases, which is expected, since this corresponds to smaller relative elongation rate and a more dominant effect of diffusion (with reduced dilution due to growth). Finally, we see that, as the inter-division time increases, the difference between the cases with and without cell division increases. This is again because increasing the inter-division time corresponds to decreasing the relative elongation rate, and thus the effect of cell division, which acts to slow down diffusion by introducing more cellular and apoplastic compartments, becomes significant. In Fig. 11b, we show a spatial profile of the GA concentration for different times. We see that, as time increases, the GA concentration initially increases, and then decreases down to zero, as in Fig. 11a. We note that, in this case, GA reaches until about a third of the length of the file. Since increasing the inter-division time reduces the relative elongation rate, it will enhance GA spreading further into the file of cells.

## Discussion and Conclusions

In this paper, we formulated a discrete cellular model for passive and facilitated hormone transport along a file of plant cells featuring apoplastic, cytoplasmic, and vacuolar compartments. We then systematically derived a continuum macroscopic representation of the model, whereby the hormone concentration depends continuously on distance along the file as well as time, exploiting the assumption that the ratio of the average cell length to the length of the whole file $\epsilon$, is small. We found that, in all cases considered, for $\epsilon \ll 1$, the hormone concentration in all cellular compartments evolves quasi-statically over the timescale associated with diffusion through the apoplast along the whole file. This enabled us to obtain a set of algebraic equations for the leading-order (in $\epsilon$) hormone concentrations in the various compartments, and we found that the hormone concentration in the apoplast and the vacuole are proportional to the hormone concentration in the cytoplasm, where the constants of proportionality form two further key dimensionless parameter groupings, that represent the ratio of the effective transport permeabilities of the hormone from the cytoplasm into the apoplast and vice versa, and from the cytoplasm into the vacuole and vice versa, respectively. These effective permeabilities incorporate the effect of passive diffusion of the protonated form of the hormone and facilitated diffusion via protein transporters of the anionic form of the hormone. In particular, for the case of GA, we found that the apoplastic and vacuolar concentrations are approximately 0.013 and 3.20 times the cytoplasmic concentration, respectively. Thus, there is very little GA in the apoplast and a significant proportion of GA is stored in the vacuole. Based on fluorescence assays, it was shown in Shani et al. (2013) that a similar ratio is measured between the vacuolar and the nuclear GA concentration. However, further experimental data are needed to fully compare our results to actual measurements.

In deriving the continuum model, we began by considering the case of identical static cells. We obtained a diffusion equation for the macroscopic cytoplasmic hormone concentration with an effective diffusivity which consisted of a cytoplasmic, apoplastic, and plasmodesmatal contribution. We considered two base cases of short ($20 \, \upmu \textrm{m}$) and long ($200 \, \upmu \textrm{m}$) cells with vacuolar volume fractions of 0.1 and 0.91, respectively, as appropriate for *Arabidopsis* root cells. We found excellent agreement between the solutions of the discrete and the continuum models for both cases even with only 20 cells. This means that we may use the continuum representation, which is much easier to solve numerically and even explicitly, to explore the dynamics of the system and investigate the effect of varying the parameters of the model. We found that, without transport through the plasmodesmata, the effective diffusivity depends on the effective hormone permeabilities across the cytoplasmic and vacuolar membranes, the geometric aspect ratios of the various compartments, and the vacuolar fraction in the cell. We observed the same functional dependence on the parameters for both short and long cells, but there was a quantitative difference in the value of the diffusivity. In the case of GA, effective diffusion in the long cells was estimated to be $80\%$ faster than in short cells, since, for a given distance, GA molecules cross cell membranes fewer times when travelling through long cells. This effect seems to dominate over the effect of having larger vacuoles in long cells, which would take up more GA, and hence reduce diffusion. Still though, the effective diffusion in both cases was significantly slower than diffusion through the apoplast, used for reference, which was due to the fragmentation of the cell into various compartments, with the apoplast being thin. We also found that transport through the continuous apoplast around all cells is significant, and, without it, the effective diffusivity was reduced 2.5 times in the case of short cells. The reduction in the case of long cells was smaller, since the proportion of the apoplast in a long cell is smaller than in a short cell.

We then explored how the effective diffusivity depended on the model parameters. For short cells, increasing the vacuolar fraction from its minimum to its maximum value decreased the diffusivity approximately three times for the case of GA. However, in a mutant plant, where there is no transporter localised on the vacuolar membrane (exporting GA out of the cytoplasm into the vacuole), the diffusivity increases with the vacuolar fraction, since this effectively decreases the relative proportion of the cytoplasm compared to the apoplast, where GA is transported faster. In fact, we obtained formulae for the effective diffusivity for loss-of-function mutants lacking a cytoplasmic importer (*npf2.12*), vacuolar importer (*npf2.14*), or both (*npf2.12 npf2.14*), and found that, in short cells, the diffusivity is largest in the double mutants and smallest in the wild type. In long cells, there was an interplay between the effect of the vacuolar importer, which was dominant in reducing the diffusivity due to the large vacuolar fraction, and that of the cytoplasmic importer, which counteracts this effect. We thus found that the diffusivity was smallest in the mutant without a cytoplasmic importer, and largest (larger by an order of magnitude) in the mutant without a vacuolar importer. When varying the geometrical parameters of the model, we observed that the effective diffusivity increased with the aspect ratio of the apoplastic compartment and increased when decreasing the aspect ratio of the cytoplasmic compartment, since both of these increase the relative proportion of the apoplast, where GA is transported fastest. We also varied the pH in the various compartments and found that the cytoplasmic pH significantly affects the effective diffusivity, with the diffusivity dramatically decreasing above a pH value of six, since this results in the cytoplasmic GA being predominantly anionic which requires transporters to mediate transport across the membranes. We found little variation in the effective diffusivity with changes in apoplastic pH and, importantly, almost no variation with the vacuolar pH. This behaviour persisted both for short and long cells, indicating that even a moderate uncertainty in the value for the vacuolar pH would not alter the tissue-scale hormone diffusion significantly. Similarly, we found that there was almost no variation of the diffusivity when varying the tonoplast transporter permeability. This suggests that it is the presence or absence of the vacuole with its transporter that alters the effective diffusivity, and not the actual value of the permeability across the tonoplast. We also found that the effective diffusivity was monotonically increasing with the passive permeability, as expected, and was monotonically increasing with the cytoplasmic importer permeability except for very small values, where it was decreasing due to a competition between its apoplastic and cytoplasmic contributions.

We also explored the effect of hormone transport through the plasmodesmata. We found that, plasmodesmatal hormone diffusion dramatically increases the effective diffusivity for both short and long cells (being comparable to the diffusivity in the apoplast), due to the direct pathway between cell cytoplasms provided by the plasmodesmata. This was also evident from the formula for the effective diffusivity with plasmodesmatal transport, where the cytoplasmic and apoplastic contributions to diffusion became negligible, and the diffusivity depended only on the permeability of the plasmodesmata and the vacuolar transporter, and the vacuolar fraction. Thus, cytoplasmic importers do not contribute to the leading-order longitudinal effective diffusion, but may contribute to transport between adjacent cell files. Thus, we see that plasmodesmata can play a significant role in hormone transport. However, the channels may be restricted by callose deposition (De Storme and Geelen 2014), so their permeability is highly varying. We conclude therefore that when they are open, plasmodesmatal transport will be dominant, even in the loss-of-function mutants; when they are closed, transport will be mediated by passive and facilitated diffusion; and when they are partially open (in the sense that the plasmodesmatal permeability is much smaller than the passive permeability of the membrane ($O(\epsilon ^2)$ in dimensionless terms)), then all three kinds of transport will contribute.

We then considered the case when the cells in the file can grow with time and be non-identical in length. While previous studies have considered cell lengths that depend on other processes, such as growth-induced forces (Jensen and Fozard 2015; Middleton et al. 2014; Murphy et al. 2019; Piatnitski and Ptashnyk 2020; Tambyah et al. 2020), here, we prescribed the dynamic cell-length, *l*(*x*, *t*), and vacuole-fraction, $\phi (x,t)$, distributions and focused on how they affected the tissue-scale transport rates. Our analysis revealed that with spatio-temporal variations in cell lengths the governing macroscopic equation for the hormone concentration is a reaction–advection–diffusion equation. In particular, both spatial variance in cell lengths (and sizes of the subcellular compartments) along the file and cell growth induced an effective advective velocity of the hormone that depended on the growth rate of the cells and the spatial gradient of the cell lengths (and the sizes of the subcellular compartments) in addition to the other model parameters. These processes also introduced an effective sink term, proportional to the hormone concentration, which represented dilution due to cell elongation. The effective diffusivity was of the same form as for the static case of identical cells, but was modulated by the cell length, which now depended on both distance along the file and time. We note that, in this case, we assumed that the cell lengths were slowly varying, which required the spatial gradient of the cell lengths to be moderate in size. Further, we neglected intracellular transport, as we assumed it happened on a fast timescale, which required that the cells did not become as long as approximately $1200 \, \upmu \textrm{m}$ when diffusion through the cytoplasm became significant. In order to better understand the individual effects of growth and spatial variance, we considered two base cases, namely, a file of identical growing cells, and a file of static cells with non-identical lengths.

In the first case, we assumed the cells grew linearly in time. We found that the effective advective velocity was entirely due to the cell growth, and we were able to remove it from the governing equation by transforming to a fixed domain, scaling the distance variable. This meant that, instead of following the actual distance along the file, we considered the hormone concentration against cell number along the file. This led to a reaction–diffusion equation, where the sink term due to dilution was proportional to the cell growth rate, and whose solutions were in excellent agreement with the solutions from the corresponding discrete model. In addition, the effective diffusivity from the unscaled equation increased with time, which was consistent with our previous results that the diffusivity was larger for long cells. The scaled formulation allowed us to find a non-uniform steady-state solution in the fixed domain. This means that, for large time, the hormone concentration in each cell will approach the corresponding steady-state value despite the fact that cells are growing. This illustrates that diffusion can balance dilution due to growth. We found that decreasing the cell growth rate made the steady-state solution more uniform, since diffusion became more dominant, whereas increasing the cell growth rate resulted in the hormone reaching only the first few cells in the file at steady state. Thus, not only is growth influenced by certain hormones, but it is also a key mechanism in regulating hormone distribution in plants.

In the second case, we assumed that the cell lengths varied linearly with distance along the file. This time, the spatial variance alone induced an effective advective velocity in the continuum equation and a sink term, whereas the effective diffusivity had the same functional form as for the growing-cells case, but it was dependent on distance, rather than time. Again, there was an excellent agreement between the solutions of the continuum and discrete models. Here, the steady state was simply a spatially uniform solution for the hormone concentration, given by the boundary condition at one end of the file. We found that increasing the gradient in cell lengths along the file (by doubling the final cell length), resulted in a larger effective diffusivity, and hence faster approach to the steady state, since diffusion is faster in long cells, as explained before. In particular, when we halved the final cell length to $100 \, \upmu \textrm{m}$, compared to the initial cell length of $20 \, \upmu \textrm{m}$, the effective diffusivity was almost uniform along the file, indicating that the cell lengths do not have to be identical to obtain a constant diffusivity. We also found a criterion for finding the direction of the induced effective velocity, which involved examining whether the ratio between the cytoplasmic and the average hormone concentration was an increasing or decreasing function with distance along the file. This resulted in positive or negative velocity, respectively. This had a significant effect on the hormone transport dynamics, as a positive velocity promoted transport along the file, whereas a negative velocity slowed it down. Further, we explored three different cases of a file made of identical cells, cells of increasing, and cells of decreasing cell lengths, respectively. To aid the comparison, we kept the number of cells, total file length and total vacuolar fraction constant among all cases. We found that, in the case of decreasing cell lengths, transport was fastest achieving half the concentration at steady state at the middle of the file two times faster than the case of identical cell lengths due to the induced positive velocity. Thus, spatial variance in cell size is a key mechanism in regulating the rate of hormone transport across the cells.

We also considered the effect of cell division on transport dynamics. We assumed that cells followed an exponential growth, which kept the relative elongation rate constant, and that each cell divided once it had doubled in size, which ensured a periodic behaviour in the cell sizes and effective diffusivity and established that the interdivision time (time between successive cell divisions) was inversely proportional to the relative elongation rate of the cells. Thus, we found that increasing the interdivision time decreased the relative elongation rate, which enhanced the effect of cell division by slowing down hormone transport when compared to the case without cell division. Similarly, this also enabled the hormone to reach further into the file of cells, as diffusion became more dominant when the growth rate decreased. In particular, for an interdivision time of 14 hours (as appropriate for cells in the *Arabidopsis* root meristem), GA diffused into approximately a third of the cell file.

Although our model was initially aimed at describing GA transport in plants, we see that the analysis is general enough to be applied to various hormones, such as ABA, auxin, SA in different regions of the plant. In fact, the framework is readily applicable to other kinds of cells, such as animal cells, where there is similar transport dynamics of a certain chemical or nutrient in a file of similar cells. The apoplastic compartment can be removed by taking its thickness to be zero and removing the corresponding concentration variables from the equations. Similarly, the vacuolar compartment can be adapted to represent a different compartment in the cell, such as the nucleus or endoplasmic reticulum, by changing the corresponding parameter values. Thus, our analysis generally demonstrates how cell-scale transport processes affect the tissue-scale hormone transport, providing formula for the effective diffusivity, effective velocity and effective dilution in terms of the cell-scale parameters. The derived continuum approximations provide key insights into hormone transport and provide a simpler macroscale representation of the hormone transport that could be incorporated into more comprehensive multiscale plant models.

## Data Availability

The Mathematica and MATLAB code used in this paper has been deposited in GitLab (https://gitlab.com/leahband/multiscaleasym_hormonetransport_growingcellfile).

## Appendix A Clarification on the Symmetry of the Cell Geometry

A typical symmetric cell that would be used as a repeating unit in multiple files of cells is a cell with horizontal apoplastic compartments on both sides with thicknesses equal to half of their original thicknesses (${\hat{a}}/2$). By symmetry, this is equivalent to considering half of this cell with a horizontal apoplastic compartment on one side of it (of thickness ${\hat{a}}/2$). This configuration corresponds to taking ${\hat{w}}$ to ${\hat{w}}/2$ and ${\hat{a}}$ to ${\hat{a}}/2$ only in the horizontal apoplastic compartments. Considering (31), we see that this transformation does not change the formula for the effective diffusivity, and thus, for simplicity, in Fig. 1, we consider a cell of width ${\hat{w}}$ with a single horizontal apoplastic compartment of thickness ${\hat{a}}$.

## Appendix B The Case of Cells with One or Two Compartments only

To illustrate the generality of our results, we use our continuum model with spatio-temporal variations in cell lengths (54)–(57c) to derive the corresponding simpler models for a file of cells that have only a cytoplasmic compartment, or a cytoplasmic compartment and an additional vacuolar or apoplastic compartment.

In the first case, we consider cells which contain only cytoplasm and have only cytoplasm-to-cytoplasm diffusion, as represented by plasmodesmatal diffusion (with $\lambda =0$, $\phi =0$, ${\mathcal {P}}_{\textrm{a}}=0$). We obtain

96 $$\begin{aligned} U_{\textrm{eff}}=u, \qquad D_{\textrm{eff}}=P_{\textrm{plas}} l, \qquad Q_{\textrm{eff}}=0. \end{aligned}$$

Thus, the effective velocity depends only on the cell growth rate. In the case of non-growing cells that have spatially varying lengths, $u=0$, so there is no induced effective velocity.

In the second case, we incorporate vacuolar compartments with facilitated transport across the vacuolar membrane (with $\lambda =0$ and ${\mathcal {P}}_{\textrm{a}}=0$). We obtain

97 $$\begin{aligned} \begin{aligned} U_{\textrm{eff}}=u-\frac{P_{\textrm{plas}} ({\mathcal {P}}_{\textrm{v}}-1) l}{(1-\phi +\phi {\mathcal {P}}_{\textrm{v}})^2} \dfrac{\partial \phi }{\partial x}, \qquad D_{\textrm{eff}}=\frac{P_{\textrm{plas}} l}{1-\phi +\phi {\mathcal {P}}_{\textrm{v}}}, \\ Q_{\textrm{eff}}=\frac{u}{l}\dfrac{\partial l}{\partial x}+\frac{1}{l}\dfrac{\partial l}{\partial t}+\frac{u({\mathcal {P}}_{\textrm{v}}-1)}{1-\phi +\phi {\mathcal {P}}_{\textrm{v}}}\dfrac{\partial \phi }{\partial x}+\frac{{\mathcal {P}}_{\textrm{v}}-1}{1-\phi +\phi {\mathcal {P}}_{\textrm{v}}}\dfrac{\partial \phi }{\partial t}-\dfrac{\partial U_{\textrm{eff}}}{\partial x}. \end{aligned} \end{aligned}$$

In the third case, we extend the first case to incorporate apoplastic compartments (but no vacuole, $\phi =0$) and consider both plasmodesmatal diffusion and facilitated transport across the cell membrane. We obtain

98 $$\begin{aligned} \begin{aligned}&U_{\textrm{eff}}=u+\frac{\lambda \omega ({\mathcal {P}}_{\textrm{a}}-1) (K(l+\lambda )+M)}{(\omega l+\lambda ( \omega +\lambda +l){\mathcal {P}}_{\textrm{a}})^2} \dfrac{\partial l}{\partial x}, \qquad D_{\textrm{eff}}=\frac{K(l+\lambda )+M}{\omega l+\lambda ( \omega +\lambda +l){\mathcal {P}}_{\textrm{a}}}, \\&Q_{\textrm{eff}}=\frac{u (\omega +\lambda {\mathcal {P}}_{\textrm{a}})}{\omega l + \lambda ( \omega + \lambda + l ) {\mathcal {P}}_{\textrm{a}}}\dfrac{\partial l}{\partial x}+\frac{\omega +\lambda {\mathcal {P}}_{\textrm{a}}}{\omega l + \lambda ( \omega + \lambda + l ) {\mathcal {P}}_{\textrm{a}}}\dfrac{\partial l}{\partial t}-\dfrac{\partial U_{\textrm{eff}}}{\partial x}, \end{aligned} \nonumber \\ \end{aligned}$$

where *K* and *M* are given by (56).

These simpler cases demonstrate that when there are additional cellular compartments, comprising either vacuoles or apoplastic compartments, then spatially varying cell lengths and vacuolar fractions create an induced effective velocity (even in the case of non-growing cells). This induced velocity is proportional to the spatial gradient of the vacuolar fraction and ${\mathcal {P}}_{\textrm{v}}-1$ in the case where vacuolar compartments are present, and is proportional to the spatial gradient in the cell lengths and ${\mathcal {P}}_{\textrm{a}}-1$, when the apoplast is included.

Furthermore, in the case of cell division with inter-division time *T* in cells with only one compartment, for example, (96) holds with *l* replaced by the modified length $l_{\textrm{d}}$ that incorporates division, where

99 $$\begin{aligned} l_{\textrm{d}}=\frac{l}{2^{\lfloor t/T \rfloor }}. \end{aligned}$$
